# Bioregion heterogeneity correlates with extensive mitochondrial DNA diversity in the Namaqua rock mouse, *Micaelamys namaquensis *(Rodentia: Muridae) from southern Africa - evidence for a species complex

**DOI:** 10.1186/1471-2148-10-307

**Published:** 2010-10-13

**Authors:** Isa-Rita M Russo, Christian T Chimimba, Paulette Bloomer

**Affiliations:** 1 Molecular Ecology & Evolution Programme (MEEP), Department of Genetics, University of Pretoria, Pretoria, 0002 South Africa; 2 Mammal Research Institute (MRI), Department of Zoology & Entomology, University of Pretoria, Pretoria, 0002 South Africa; 3 DST-NRF Centre of Excellence for Invasion Biology (CIB), Department of Zoology & Entomology, University of Pretoria, Pretoria, 0002 South Africa; 4 Cardiff School of Biosciences, Biomedical Sciences Building, Cardiff University, Museum Avenue, Cardiff, CF10 3AX UK

## Abstract

**Background:**

Intraspecific variation within the diverse southern African murine rodents has not been extensively investigated, yet cryptic diversity is evident in several taxa studied to date. The Namaqua rock mouse, *Micaelamys namaquensis *Smith, 1834 is a widespread endemic murine rodent from the subregion. Currently, a single species with four subspecies is recognised, but in the past up to 16 subspecies were described. Thus, this species is a good candidate for the investigation of patterns and processes of diversification in a diverse but under-studied mammalian subfamily and geographic region. Here, we report genetic differentiation based on mitochondrial DNA (mtDNA) cytochrome *b *(cyt *b*) sequences among samples collected over an extensive coverage of the species' range.

**Results:**

Cytochrome *b *sequences of 360 widely sampled individuals identified 137 unique maternal alleles. Gene tree and phylogeographic analyses of these alleles suggest the presence of at least eight lineages or haplogroups (A-H), with varying degrees of intra-lineage diversity. This differentiation is in contrast with the most recent taxonomic treatment based on cranial morphometrics which only recognised four subspecies. The mtDNA diversity strongly supports earlier views that this taxon may represent a species complex. We further show statistical support for the association of several of these lineages with particular vegetation biomes of southern Africa. The time to the most recent common ancestor (TMRCA) dates to the Pliocene (~5 Mya) whereas coalescent-based divergence time estimates between lineages vary between 813 Kya [0.22 - 1.36] and 4.06 Mya [1.21 - 4.47]. The major diversification within lineages occurred during the Pleistocene. The identification of several regions of sympatry of distinct lineages offers future opportunities for the elucidation of the underlying speciation processes in the suggested species complex.

**Conclusions:**

Similar to other African murine rodents, *M. namaquensis *radiated during the Pliocene and Pleistocene coinciding with major periods of aridification and the expansion of savanna habitats. The suggested species complex is represented by at least eight lineages of which the majority are confined to only one or a few neighbouring biomes/bioregions. Contrasting intra-lineage phylogeographic patterns suggest differences in adaptation and responses to Plio-Pleistocene climatic and vegetation changes. The role of ecological factors in driving speciation in the group needs further investigation.

## Background

Globally the order Rodentia represents the richest mammalian diversity [1], yet species and higher level classification within the order remains to be resolved due to controversies surrounding morphological character evolution in the group [2-5]. The Old World subfamily Murinae is the most diverse within the species-rich family Muridae [3]. Africa is one of the centres of endemism of the subfamily and based on combined mitochondrial and nuclear gene data, it has been suggested that the major African radiation occurred in the Miocene (7 - 9 Mya) [3]. The latter and other recent studies [6,7] of African murids highlighted the impact of Miocene and Pliocene climatic changes on vegetation and landscape composition (see also [8]) and linked the rapid radiation within several taxa to the expansion of savanna habitats.

Murid diversity within the southern African subregion remains underestimated as few local taxa have been assessed for intraspecific variation. Most of the earlier studies were based on traditional morphometrics and/or qualitative morphology, e.g. studies on species within the genera *Acomys *[9], *Saccostomus *[10], *Mastomys *[11], *Aethomys *[12-15] and *Micaelamys *(formerly designated as *Aethomys *[2]). Recently molecular studies investigated *Otomys *[16], *Mastomys *[17,18], *Micaelamys *[19], *Rhabdomys *[20] and *Saccostomus *[21]. These molecular studies suggest high levels of cryptic diversity, a feature also reported for other African murids (cf [6,7]).

In general, small mammals such as rodents have restricted dispersal abilities [22-24] and many display patchy distributions. Smaller rodents in particular show adaptation to specific micro-habitats and would likely be more sensitive to environmental changes [24,25]. Indeed, habitat selection, dispersion and inter-specific competition are proposed to be amongst the most important factors influencing the co-existence of species [23,26]. In addition, karyotypic changes have been implicated in the speciation of several rodent species (e.g., *Otomys *[27] and *Rhabdomys *[20]).

The focus of the present study is the Namaqua rock mouse, *Micaelamys namaquensis *Smith, 1834, originally described from Witwater, Little Namaqualand in the Northern Cape Province of South Africa [28]. The genus *Aethomys *Thomas, 1915 was formerly subdivided into two African endemic subgenera namely *Micaelamys *and *Aethomys *[2]. Recent molecular studies [19,29,30] reported the paraphyly of the genus and the two subgenera have since been elevated to full generic rank [2]. The genus *Micaelamys *Ellerman, 1941 includes *M. namaquensis *and *M. granti *Wroughton, 1908, while *Aethomys *includes the remaining nine species. The close relationship between *M. namaquensis *and *M. granti *is evident from dental morphology [31], karyology [32], gross sperm and bacular morphology [32] and cranial phenetic analysis [33].

Fossil species of *Aethomys *have been recorded from Langebaanweg (Western Cape Province, South Africa) [34]. It is noteworthy that the smaller of the two fossil species closely resembles the extant *M. namaquensis*, which may be indicative of the long-term presence of this species in the subregion [34]. The oldest known representatives of the genus, *A. adamanticola *and *A. modernis*, have been recorded in South Africa and date to between the Early Pleistocene and Late Miocene [35,36].

*Micaelamys namaquensis *is widely distributed in southern African (south of the Zambezi/Cunene Rivers), but has also been recorded to the north of the subregion in Angola, Zambia, Malawi and northern Mozambique [2]. These rodents are catholic in their habitat requirements but where there are rocky outcrops or hillsides they will use these in preference to any other type of habitat [2]. The social structure of the species has not been elucidated but colonies appear to live in rock crevices, in or under fallen logs or in holes in trees [2]. They are mostly granivores [2] and breed during the warmer spring and summer months [37]. They are known for unstable population cycles associated with high mortality and high reproductive potential [38]. Such cycles would likely leave clear 'footprints' in the distribution of genetic diversity in the species.

**Figure 1:**
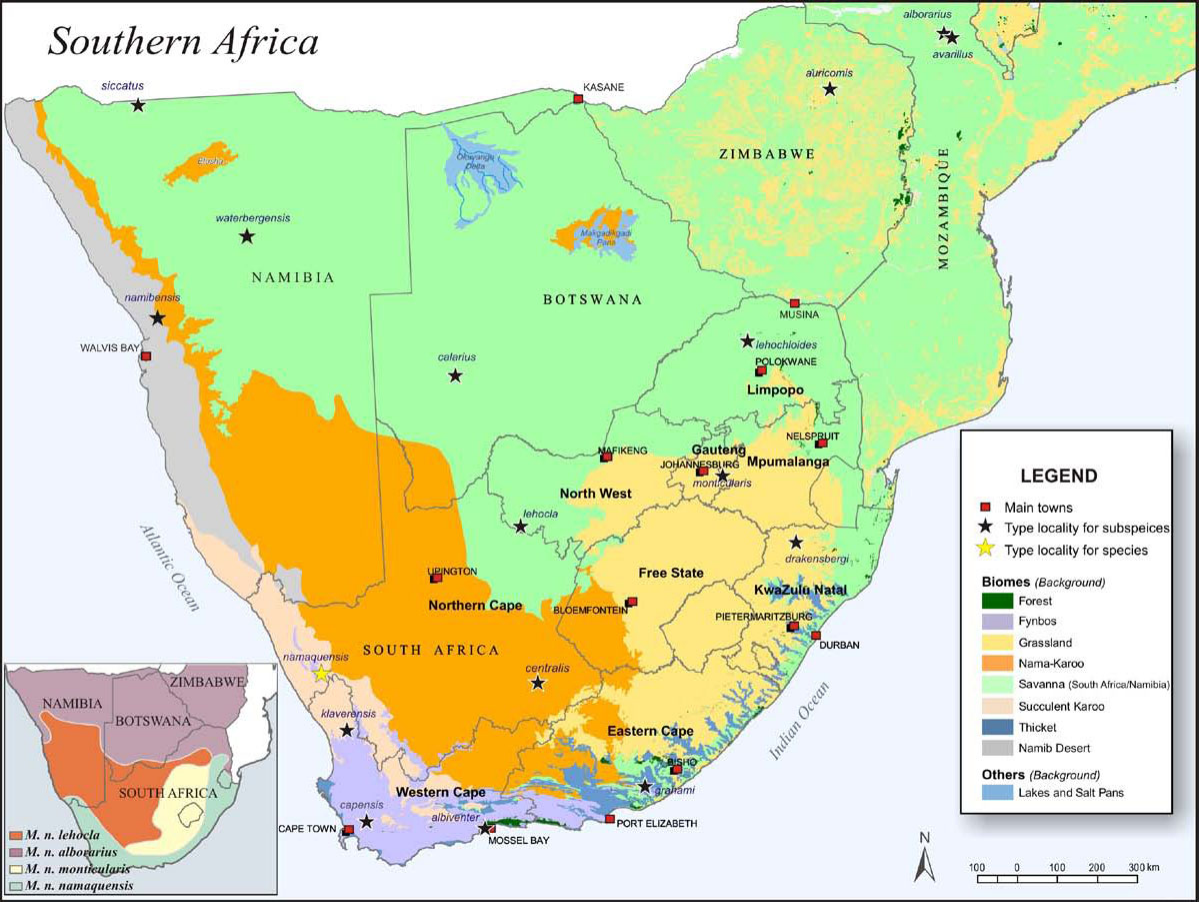
**Major biomes of southern Africa [see **[44,45]**]**. Background colours for the eight major biomes are indicated in the legend; major lakes and saltpans are also included. The yellow star indicates the type locality for the species and the blue stars indicate the type localities for the 16 previously described subspecies. The inset shows the geographic distribution of the phenetic diversity within *Micaelamys namaquensis *from southern Africa [15]. The purple, orange, yellow and green zones correspond with the subspecies *M. n. alborarius*, *M. n. lehocla*, *M. n. monticularis *and *M. n. namaquensis*, respectively.

The Namaqua rock mouse shows considerable geographic variation such as in pelage colouration, tail length and body size throughout its distributional range [2,37,39,40]. This variation suggested that *M. namaquensis *may reflect either a complex of species [40] or subspecies [37,39]. Earlier reports [41,42] recognised 16 subspecies within *M. namaquensis *(Figure 1). However, these distinctions were primarily based on a limited number of geographically restricted samples [37,43], with little or no assessment of patterns of geographic variation over the entire distributional range of the species.

A comprehensive intraspecific morphometric study within *M. namaquensis *from southern Africa [15] suggested the recognition of four subspecies differing in both cranial size and shape: *M. n. namaquensis *Smith, 1834, *M. n. lehocla *Smith, 1836, *M. n. alborarius *Peters, 1852 and *M. n. monticularis *Jameson, 1909. This study also suggested that the geographical limits of the proposed subspecies broadly coincide with the major phytogeographical zones of southern Africa [44,45] (Figure 1): the subspecies *namaquensis *was shown to be largely associated with a combination of the Succulent Karoo, Fynbos and the southern coastal Savanna/Grassland region of the Eastern Cape, KwaZulu-Natal and eastern Mpumalanga Provinces of South Africa, while the subspecies *alborarius *and *lehocla *were shown to be associated with the Savanna and Upper/Lower Karoo biomes, respectively [15]. The subspecies *monticularis *was largely confined to the Grassland biome of southern Africa [15] (see inset in Figure 1).

Given its wide distribution and past indications of cryptic diversity, we investigated the cyt *b *variation of *M. namaquensis *as a potential model for murine diversification in southern Africa. Our objectives are: 1) to evaluate the phylogeographic structure in this widespread species; 2) to compare patterns of genetic variation with previously described morphological variation; 3) to evaluate the geographical association between the observed diversity and environmental features of the region and 4) to consider the divergence times in terms of the climatic and vegetation changes characterising southern Africa over the recent evolutionary past.

## Results

The 5' end of the cyt *b *gene (631 bp) was sequenced for 360 *M. namaquensis *individuals. All sequences obtained were of the mtDNA cyt *b *gene with conserved domains and nucleotide composition biases typical of this mtDNA protein coding gene [46-48]. As expected, most of the substitutions were silent, with only 36 of the 210 amino acid sites being variable. The 176 variable nucleotide positions defined 137 maternal alleles (Additional file 1), the allele frequencies and geographic distributions are indicated in Additional file 2. Sequence divergence values based on uncorrected p-distances ranged from 0.16% to 7.74% between the alleles. Only four widespread and high frequency alleles were recorded (NH014, NH100, NH113 and NH115), while most of the remaining alleles were locality-specific (107 alleles). The overall nucleotide diversity was 3%, while the allelic diversity value of 0.91 corresponded to those reported for other rodents [49].

**Figure 2:**
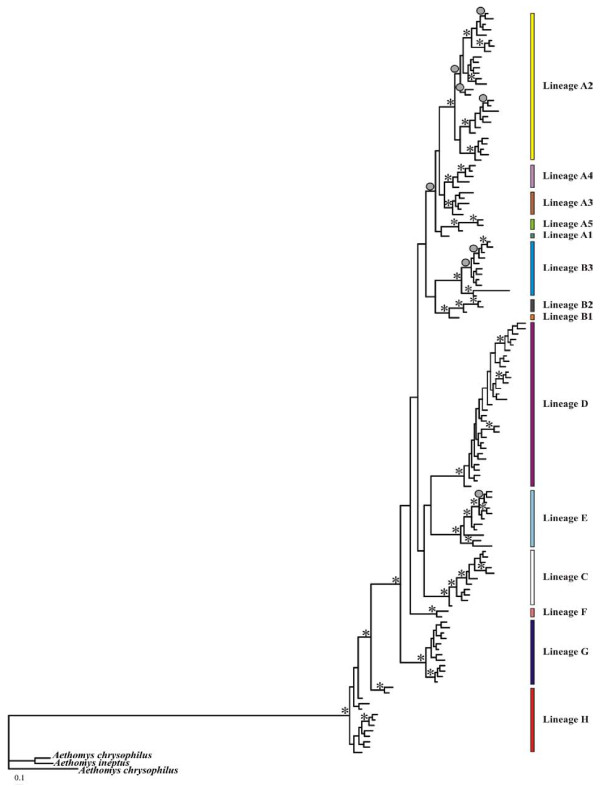
**A Bayesian inference (BI) tree based on 631 bp of the mitochondrial DNA (mtDNA) cytochrome *b *(cyt *b*) gene of *Micaelamys namaquensis *from southern Africa**. The BI posterior probability values for internal branches are given at each node with either an asterisk (*) or a circle (•). Asterisks indicate BI posterior probability values ≥ 0.95 and circles indicate BI posterior probability values ≥ 0.90. *Aethomys chrysophilus *and *A. ineptus *were used as outgroups. Lineages A - H mainly correspond to different biomes or bioregions of southern Africa (Figures 1 and 5; see text for some minor exceptions). Colours correspond to those in Figures 3, 4 and 5.

**Figure 3:**
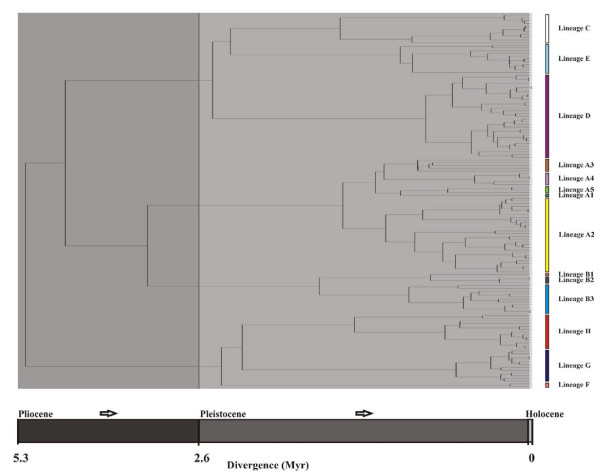
**A phylogenetic tree for 137 representative mitochondrial DNA (mtDNA) cytochrome *b *(cyt *b*) alleles of *Micaelamys namaquensis *from southern Africa as obtained by BEAST analysis**. Divergence dates (epochs) are indicated in the grey-scale key at the bottom of the figure. Lineages A - H mainly correspond to different biomes or bioregions of southern Africa (Figures 1 and 5; see text for some minor exceptions). Colours correspond to those in Figures 2, 4 and 5.

**Figure 4:**
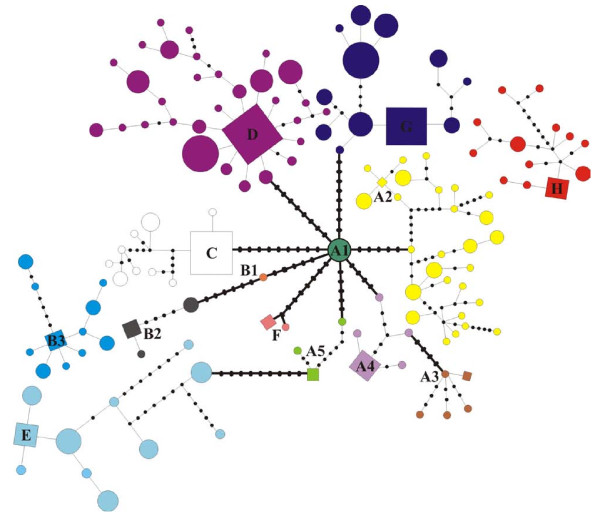
**Minimum-spanning and TCS networks indicating the least number of mutational steps between composite mitochondrial DNA (mtDNA) cytochrome *b *(cyt *b*) alleles within *Micaelamys namaquensis *from southern Africa**. Sizes of the circles and squares represent allele frequencies; each connecting line represents a mutational step and solid black circles indicate un-sampled/extinct alleles (TCS analysis only connected alleles at the 95% confidence limit, i.e., 10 steps). Squares represent potential ancestral haplotypes as identified by the TCS analysis. Thick black lines and thinner black lines indicate connections between groups and within groups, respectively.

Unique lineages were defined based on the joint interpretation of a Bayesian gene tree (Figure 2), an ultrametric analysis in BEAST (Figure 3) and an allele network as estimated in TCS (Figure 4) (see Additional file 3 for a summary of the comparison). We thus propose the presence of eight lineages (A-H) with varying degrees of intra-lineage diversity. We do not present individual lineage phylogeographic patterns in detail but indicate some of the subdivisions (e.g. in lineages A and B) and use lineages A2 and D to illustrate heterogeneous and homogeneous diversity patterns, respectively.

The Bayesian phylogram (Figure 2) is characterised by short internal branches suggesting a rapid radiation of lineages. This is supported by the relatively short timeframe over which these lineages became established during the Early Pleistocene and Late Pliocene (2.3 - 4.06 Mya) (Figure 3). Two major periods of subsequent radiation within the lineages is evident at approximately 1.4 - 1.7 Mya (diversification within lineages A, B, C and H) and from 176 Kya to 1 Mya (lineages D - G).

With the exception of lineage H, the monophyly of the respective lineages are statistically supported by the Bayesian posterior probability values (Figure 2). Sister relationships are not consistent across the phylogenetic analyses and statistical support is largely lacking (see Figures 2 and 3 and Additional file 3). However, lineages A and B and lineages C, D and E are potential sister clades. In addition, these two clades appear to share a common history. The BEAST analysis further suggest a sister relationship between G and H; these two distinctive lineages are consistently placed sister to the remainder of the lineages. The position of lineage F is inconsistent between the different analyses.

The TCS analysis connected alleles separated by up to 10 mutational steps (95% confidence limit); only lineage H and a subset of the lineage B alleles (B3) could not be joined to the remainder of the network (Figure 4). Lineage D shows a star-like pattern of 31 closely related alleles recorded over a large geographic area. Seven of the alleles were shared between two to six localities; these included both ancestral and derived alleles, indicative of retention of ancestral polymorphisms as well as more recent expansions of this lineage. In contrast, most of the other lineages harboured more divergent alleles, as evident by the presence of intervening, extinct or un-sampled alleles. For example, lineage A2 was characterised by six alleles that were shared between localities while the remaining alleles were locality specific. Some of these shared alleles were more derived but some (NH45, NH46, NH51) were still ancestral. The nucleotide and haplotype diversity estimates within the lineages are shown in Table 1. Lineages A5 and F showed the highest within lineage sequence divergences (0.35% and 0.65%, respectively).

**Table 1:** Mitochondrial DNA (mtDNA) cytochrome *b *(cyt *b*) sequence divergence, nucleotide and haplotype diversity values between and within lineages/haplogroups as identified by MINSPNET, TCS and phylogenetic analyses, within *Micaelamys namaquensis *from southern Africa.

GROUPS/LINEAGES	UNCORRECTED P-DISTANCE WITHIN LINEAGE DIVERGENCE	UNCORRECTED P-DISTANCE BETWEEN LINEAGE DIVERGENCE	HAPLOTYPE DIVERSITY	PERCENTAGE NUCLEOTIDE DIVERSITY
Lineage A1	-	0.84 - 3.69%	-	-

Lineage A2	0.16 - 2.81%	1.15 - 6.15%	0.97	1.44

Lineage A3	0.17 - 1.21%	0.84 - 5.18%	1.00	0.62

Lineage A4	0.18 - 1.22%	0.88 - 5.03%	0.79	0.46

Lineage A5	0.35 - 1.58%	1.34 - 5.85%	0.83	0.89

Lineage A1-A5	0.16 - 4.46%	1.62 - 6.15%	0.98	2.11

Lineage B1	-	1.18 - 4.26%	-	-

Lineage B2	0.16 - 0.97%	1.18 - 5.63%	0.80	0.56

Lineage B3	0.16 - 3.31%	2.48 - 7.74%	0.90	0.73

Lineage B1-B3	0.16 - 4.63%	1.62 - 7.74%	0.94	1.68

Lineage C	0.16 - 2.06%	2.35 - 6.35%	0.55	0.57

Lineage D	0.16 - 1.81%	2.47 - 7.74%	0.81	0.37

Lineage E	0.16 - 2.96%	2.19 - 6.63%	0.86	0.99

Lineage F	0.65%	1.99 - 5.29%	0.67	0.45

Lineage G	0.16 - 1.17%	2.04 - 6.61%	0.69	0.35

Lineage H	0.16 - 2.10%	3.01 - 6.47%	0.93	0.63

Lineage letter codes as defined in this table are used in all other Figures, Tables and Additional files.

**Figure 5:**
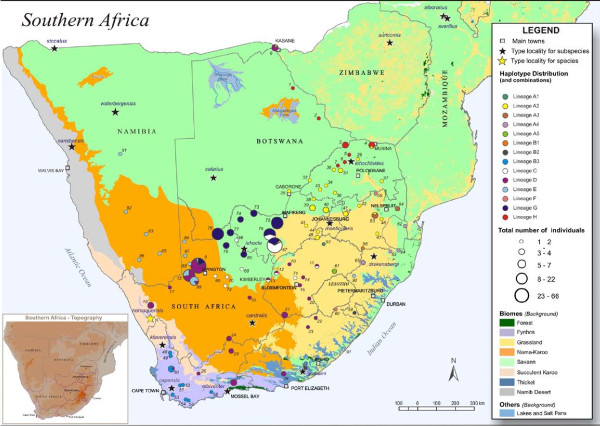
**Geographic distribution of *Micaelamys namaquensis *mitochondrial DNA (mtDNA) cytochrome *b *(cyt *b*) lineages in the biomes of southern Africa (see legend for colours)**. The circles indicate the number of individuals sampled at each locality (see legend for scale); the size of the pie charts represents allele frequencies. Stars indicate the type localities of the species/subspecies. The inset shows a topographical map of southern Africa. Colours correspond to those in Figures 2, 3 and 4.

Most lineages form separate geographical units displaying an allopatric/parapatric pattern of distribution (Figure 5). Major physical features of the southern African landscape (rivers and mountains) do not appear to separate the lineages (see inset Figure 5 for the general topography). One exception is the enigmatic lineage F that appears to be restricted to high elevations of the Great Escarpment. However, most lineages and in some instances also the sub-clades within them, appear to be associated with different vegetation types of southern Africa: lineage A (A1 - A5) with different bioregions of the Grassland and Savanna biomes; B2 with Albany Thicket; B3 with the western Fynbos; C with the Bushmanland/Upper Karoo bioregion (Nama-Karoo/Savanna); D with the Nama-Karoo; E with the Kalahari Duneveld (Nama-Karoo); F with the Sub-Escarpment Grassland bioregion (Grassland); G with the Eastern Kalahari Bushveld (Savanna); and H with Savanna. Names in parenthesis (for example Nama-Karoo/Savanna) refer to biomes as indicated in Figure 1. For bioregion names not referred to in Figure 1 and for more detailed maps see [45].

In addition to the distributions above, lineages D and E were also sampled from geographically distant localities (locality 8 for lineage D and localities 90, 91 for lineage E). Lineage A5 also has a disjunct distribution including individuals from Fouriesburg (Free State Province, South Africa) and Kasane (Botswana) which are approximately 1 000 km apart. Lineages A1 and B1 were only recorded from single localities, Koppies Dam Nature Reserve (Free State Province, South Africa) and Volksrust (Mpumalanga Province, South Africa), respectively (Figure 5). Several of the lineages were found in sympatry (Figure 5), for example, lineages A2, C and D were recorded with several other lineages.

**Figure 6:**
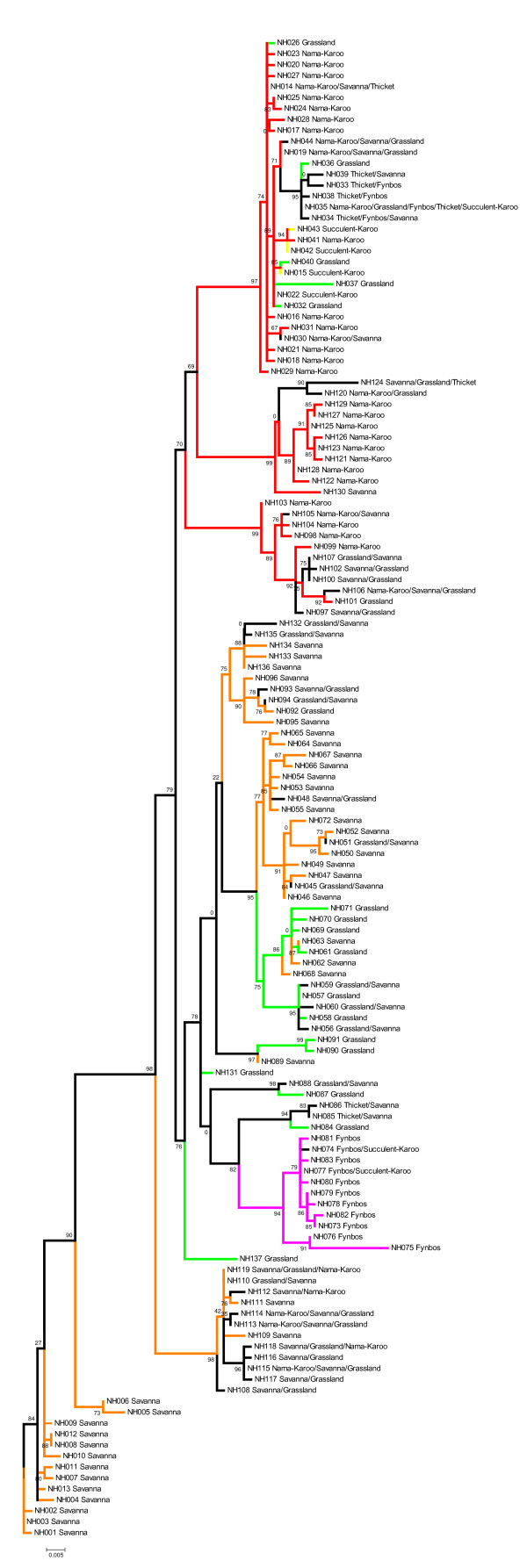
**Maximum likelihood (ML) reconstruction of the 137 maternal alleles identified for *Micaelamys namaquensis *using the online version of PhyML.** This tree was reconstructed to test for association between lineages (phylogeographic patterns) and biomes. Support for each node was assessed with 100 bootstrap replications. Different colours represent support for the different biomes: green = Grassland biome; red = Nama-Karoo biome, pink = Fynbos biome and orange = Savanna biome.

There is good support for the association between lineages and the major biomes of southern Africa (see Figure 1) as shown by the ML phylogram (Figure 6). Bootstrap values are indicated and different colours correspond to different biomes (see figure legend for details). Alleles within most lineages are associated with single biomes (e.g. haplotypes NH001 - NH012 of lineage H with Savanna; haplotypes NH073 - NH083 of B3 with Fynbos) or two neighbouring biomes (e.g. all alleles within lineage A). Although its core association is with the Nama-Karoo biome, lineage D is geographically the most widespread (see Figure 5) across several neighbouring biomes.

Figure 5 further allows a comparison between the geographic distribution of previously recognised subspecies and the distribution of mtDNA cyt *b *diversity. Four of the mtDNA lineages (lineages A2, B3, D and H) broadly correspond with the biome-related distributional patterns of the subspecies *monticularis*, *namaquensis*, *lehocla *and *alborarius*, respectively proposed by [15] (see also inset in Figure 1). However, there seems to be a better correspondence to the formally described subspecies [41,42] (see Figure 1).

A Mantel nonparametric test for lineages identified in the phylogenetic and phylogeographic analyses (see Additional file 4 for variation) revealed isolation by distance for four of the clades (*p*-values indicated in bold, see Additional file 4). In most instances, the standard normal variate (*g*) was smaller than the critical value of 2.575 at *P *≤ 0.005, indicating that the null-hypothesis (no association between elements in the two matrices) could not be rejected. Mantel tests could not be performed for lineages represented by too few individuals.

## Discussion

The analysis of the mtDNA cyt *b *gene in the present study supports previous suggestions that *M. namaquensis *from southern Africa is polytypic [15,19,41,42]. The present analysis, however, reveals a much higher degree of variation, comparable with other small mammalian taxa [6,50-52]. This is in contrast to a morphometric study that proposed the recognition of only four subspecies within *M. namaquensis *[15]. The detection of partial overlap between intra-lineage and inter-lineage divergences suggests the need for combined analyses with nuclear genes and more detailed morphological/behavioural data to fully resolve species-level diversity and the biogeographic history of this group of murid rodents that may represent a species complex.

Levels of variation seen here are comparable to those of other within-population or within-species comparisons in muroid rodents. For example, sequence divergence within Andean mice of the genus *Akodon *ranged between 7.3 and 11.7% [53]. There is currently no consensus as to the sequence divergence level associated with particular taxonomic ranks [54]. While [55] suggested that values of 4% reflect inter-specific differences in rodents, [56] advocated values higher than 11% at this level. [57] cautioned against the use of sequence divergence levels in assigning specific status. Possible problems may also arise from interpreting evolutionary history entirely on the basis of one gene. However, it is significant that some of the lineages identified in the present study have previously been proposed as subspecies on classical morphological/morphometric grounds [15].

Consideration of *M. namaquensis *diversity within a biological species concept (BSC) framework would require accurate delineation of the distributional boundaries of the identified lineages and mapping of areas of overlap through further sampling and genetic analyses. In areas of either sympatry (e.g. localities 4, 8, 9, 11, 12, 13, 16, 50, 67, 79, 89 and 93) or parapatry (e.g. localities 5 - 7 and 29 - 31), the presence or absence of hybrids (identified with diagnostic nuclear or cytogenetic characters) could be used to test the breeding integrity of these groups. Breeding isolation has for example, been shown between *A. chrysophilus *and *A. ineptus *using karyology and sperm and bacular morphology [32,58-60].

The remarkable karyotypic diversity found in small mammals suggests that speciation may be associated with karyotypic changes [61]. This is certainly true for some rodents that include variation in diploid numbers [32,60,62,63]. Other examples include southern African rodent species within the genera *Mastomys*, *Saccostomus *and *Otomys *where morphologically similar but genetically distinct species complexes have been identified using karyotypes and/or protein electromorph mobility [60,61]. A single diploid number (2*n *= 24) has thus far been reported in *M. namaquensis *[32] however, karyotypic variation, in addition to locally adaptive physiological and/or behavioural responses [64-67] may have played a significant role in speciation.

Three major periods of diversification were identified in the present study: 2.03 - 4.06 Mya (the origin of lineages A - H), 1.25 - 1.7 Mya (deeper divergences within lineages A, B, C and H), and 176 Kya to 1 Mya (radiation within the rest of the lineages). The most significant time for *M. namaquensis *diversification therefore corresponds to the Pleistocene and Pliocene, a period characterised by major climatic and vegetation changes with direct and indirect impacts on animal populations [8,64].

The Early Pleistocene and Pliocene were characterised by uplifts which raised the interior plateaus of South Africa more than 1 800 m above the Miocene level [65]. Between 5 and 6.5 Mya, a severe temperature drop (the Terminal Miocene Event) resulted in a rapid and dramatic sea level drop of over 100 m worldwide. The temperature during the subsequent period appeared to have fluctuated until between 2.5 - 2.6 Mya. Since then, temperatures have oscillated between glacial and interglacial conditions at least 17 times during the last 1.7 million years with individual cycles having a mean duration of about 100 000 years [66].

These temperature changes had a definite impact on vegetation and may have contributed to speciation in southern African mammals [8,67]. The three major periods of diversification of *M. namaquensis *lineages correspond to periods of aridification (2.4 - 2.9 Mya; 1.6 - 1.8 Mya and 0.8 - 1.2 Mya) that also affected the southern African environment [8]. These arid periods alternated with wetter conditions so that populations were shifting continuously [67]. These cycles could have resulted in speciation, either through physical isolation (allopatric speciation) or changes in habitat (sympatric or ecological speciation).

The diversification within the *M. namaquensis *species complex also coincides with the timeframe proposed for the rapid radiation within the African Murinae [3]. Current fossil evidence also suggests that representatives of the genera *Aethomys *(*A. modernis*, *A. lavocati, A. deheinzelini*) and *Micaelamys *(*A. adamanticola*) were present in southern Africa since the Late Miocene and one of these fossils closely resembles the extant *M. namaquensis *[34-36]. The expansion and contraction of savanna habitats across especially the eastern and southern parts of Africa during the Pliocene and Miocene have been linked to speciation in several other rodent genera, for example *Tatera *[68], *Hylomyscus *[7] and *Praomys *[25]. Based on the timing and pattern of diversification in *M. namaquensis *and these other broadly co-distributed rodents, we propose that vicariance, dispersal and local adaptation shaped the diverse rodent fauna of the continent and likely also the southern African subregion.

The chronology (Figure 3) and habitat association (Figure 6) presented here suggest that the initial radiation within *M. namaquensis *was in open habitats (Savanna and Grassland biomes) in the northern parts of southern Africa and that one of these ancestral lineages persisted in the drier savanna regions (giving rise to lineages G and H). The sister lineage diverged into several arid-adapted lineages mostly centred on the Savanna and Nama-Karoo biomes (lineages C, D and E) while the remaining lineages adapted to more mesic habitats (interior savanna/grassland, fynbos and coastal habitats; lineages A and B). The origin and affinities of lineage F, recorded from mesic grasslands of the Great Escarpment, remain unresolved.

The extant distributions of some of the lineages remain puzzling. For example, the presence of lineage E at Bergville, KwaZulu-Natal Province (locality 90; Figure 5, Additional file 2, 5 and 6) and Dwesa Nature Reserve, Eastern Cape Province (locality 91; Figure 5, Additional file 2, 5 and 6) may be a result of recent Kalahari sand flows from north to south [69,70]. Pleistocene sand deposits have been recorded from the Eastern Cape Province above the Great Escarpment at an altitude of 1 800 m (north of Queenstown, Eastern Cape Province: 31°23"S; 26°41"E) [71]. Other sand sheets have been reported from the western Free State, Gauteng and Northern Cape Provinces [72]. As a species' habitat expands or shifts, so does the distributional range of that species [69]. This may also be the case with the occurrence of lineage D at Kasane (locality 8; Figure 5, Additional file 2, 5 and 6) in Botswana. Some studies [73,74] have suggested incomplete mtDNA lineage sorting and the retention of ancestral polymorphisms as possible explanations.

Habitat changes likely also played a key role in shaping the phylogeographic structuring within lineages. Radiation within lineages did not occur at the same time and it is clear from Figures 3 and 4 that different processes have contributed to the levels of diversity within lineages. As has been reported for other African mammals [64] different refugia may have existed in unfavourable climatic conditions and acted as sources for subsequent range expansions. The typical population cycles within *M. namaquensis *[38] and meta-population dynamics would further have contributed to episodes of extinction and recolonisation.

Lineage A serves as an example of one of the lineages with deep phylogeographic subdivision dating to the mid-Pleistocene (~1.51 Mya). The population history within lineage A2 has been more complex, potentially due to waves of colonisation or dispersal among regions associated with the expansion of grassland and savanna habitats. It is possible that large population sizes of lineage A2 are responsible for maintaining both the high nucleotide diversity (1.44%; Table 1) and the divergent alleles. Six alleles were shared between localities and the presence of several unique alleles within localities may reflect evolutionarily old populations, which may have served as core regions for colonisation.

It has been proposed that Grassland expanded during the glacial periods due to a colder climate that allowed for the formation of frost [75,66]. If the association between Grassland and winter frost, together with the added effects of fire is significant, it is plausible to suggest that when the area of winter cold expanded periodically in the past, it may have caused an expansion of grassland at the expense of other vegetation types. There is evidence from Aliwal North (Eastern Cape Province) and Florisbad (Free State Province) that during the last glacial maximum (LGM), grassland replaced other vegetation types [76,77]. The genus *Lepus *is a recent arrival in southern Africa associated with the expansion of grassland over the past million years. Subsequent habitat changes have for example, generated distinct scrub hare, *L. saxatilis *phylogroups in the central and northern regions of South Africa [78]. It would be instructive to compare the observed pattern of *M. namaquensis *in the grassland to other co-distributed small mammal species, as congruence in phylogeographic patterns between independent lineages would reflect similar vicariant events [79].

At the other extreme, a star-like pattern of closely related alleles is evident in lineage D, despite large geographic distances between localities (Figure 5). Rare alleles are more likely to be mutational derivations of the common alleles [80]. Therefore, the presence of the closely related alleles within almost all localities within lineage D reflects a common female ancestry, probably due to recent range expansion from a source population(s). In essence, lineage D exhibits limited phylogeographic structure with a relatively low nucleotide diversity (0.37%; Table 1). Similar patterns of low genetic structuring have been recorded for other small mammals [81-84]. Therefore, different evolutionary processes have been involved shapping genetic diversity within the different lineages.

## Conclusion

*Micaelamys namaquensis *displays considerable mtDNA diversity in contrast with the current taxonomic view based on morphology/morphometrics. Taken together with previously reported geographic variation in fur colour, tail length and body size we believe that the taxon represents a species complex in southern Africa. Given the strong geographic association of most of the eight identified lineages with specific biomes or bioregions our future research will explore processes underlying ecological speciation in the group.

## Methods

Samples (*n *= 360) representative of the four previously proposed morphometrically-defined subspecies [15], from 95 localities spanning the four major phytogeographical zones in southern Africa, were collected from South Africa, Namibia, Swaziland and Botswana (see Additional files 2, 5 and 6). Only areas near type localities of six of the originally described subspecies (*waterbergensis*, *calarius*, *siccatus*, *auricornis*, *alborarius *and *avarillus*; Figure 1) from northern Namibia, western Botswana, Zimbabwe and Mozambique, were not sampled (Additional file 6). Animals were live-trapped using Sherman traps (H.B. Sherman Traps Inc. Florida, U.S.A.) and handled under the guidelines of the American Society of Mammalogists (ASM; http://www.mammalogy.org/committees/index.asp; Animal Care and Use Committee, 1998) as approved by the Animal Ethics Committee of the University of Pretoria (Project number: EC 010417-004). Collection permit numbers are given in Additional file 7. Some animals were sacrificed by halothane inhalation, and ear clips from the rest were either frozen at - 20°C, stored in 70% EtOH, or in Tissue/Blood Storage Buffer (100 mM Tris, 40 mM EDTA, 1 M NaCl and 0.5% SDS). Voucher specimens were prepared using standard natural history museum procedures for small mammals and were deposited in the mammal reference collection of the Transvaal Museum (TM) of the Northern Flagship Institution (NFI), Pretoria, South Africa.

Total genomic DNA was extracted from tissue using either a standard phenol/chloroform protocol [85] or a Sigma GenElute™ Mammalian Genomic DNA Miniprep Kit (Sigma Aldrich). Some problem samples were extracted using a Genomic DNA Mini Kit I Multi (Animal/Plant) (Koma Biotech Inc.).

A mouse-specific primer (H15309) was designed in the tRNA-Thr from a *Mus musculus *sequence [GenBank: J01420; [86]]. This primer is a *M. musculus *version of H15915 [47] and was used in combination with the shortened universal vertebrate primer L14724 [87] that anneals in tRNA-Glu, to amplify the cyt *b *gene of two *M. namaquensis *individuals. An *A. chrysophilus *sequence [GenBank: AF004587; [50]], together with these *M. namaquensis *sequences were aligned in Clustal X [88] for designing an internal species-specific *M. namaquensis *primer (H14769, 5' GTCTGCGTCTGAATTTAG 3'). H14769 was used in combination with the shortened L14724, or L14841 of [89], to amplify the 5' end of the cyt *b *gene for all individuals in our study. A preliminary analysis showed that the 5' end of the cyt *b *gene yielded considerable levels of variation within *M. namaquensis *and a 631 bp region was used in subsequent analyses.

Polymerase chain reactions (PCR) [90] were performed in a total volume of either 50 μl or 25 μl. Reactions contained approximately 50 - 100 ng genomic DNA template, 1 × buffer, 2.5 mM MgCl_2_, 0.2 mM of each of the four nucleotides (Promega), 2.5 - 5 pmol of each primer and 0.15 U of Super-Therm^® ^DNA polymerase (Southern Cross Biotechnology). PCR conditions were as follows: denaturing at 94°C for 5 minutes, 35 cycles of the following: 94°C for 30 seconds, primer annealing at 52°C for 30 seconds and elongation at 72°C for 45 seconds. This was followed by an extended elongation step for 7 minutes at 72°C in a Geneamp^® ^PCR System 9700 (Applied Biosystems). The PCR products were purified using the High Pure™ PCR Product Purification Kit (Roche Diagnostics). Dye-terminator cycle sequencing was performed for both the light and heavy strands using the ABI PRISM Big Dye™ Terminator version 3.1 Cycle Sequencing Ready Reaction Kit (Applied Biosystems). Cycle sequencing products were subsequently precipitated using a NaAc salt method (Applied Biosystems). Nucleotide sequences were determined using an ABI 3130 automated sequencer (Applied Biosystems).

The quality of the raw sequence data was evaluated in Sequencing Analysis, version 3 (Applied Biosystems) or BioEdit, version 7.0.9.0 (Ibis Biosciences), and a consensus sequence for each individual from forward and reverse sequences was determined in Sequence Navigator, version 1.01 (Applied Biosystems) or Vector NTI Advance 10 (Invitrogen). All sequences were deposited [GenBank: GQ471959 to GQ472095]. These accessions represent all unique haplotypes identified in the present study, including geographical information. Consensus sequences of all individuals were aligned in Clustal X [88].

We used the results of all phylogenetic and phylogeographic analyses to define unique lineages (see also [91]). Only lineages/haplogroups with support in at least two of the three analyses were considered as separate lineages while other subdivisions were treated as variation within these lineages. Further research will test the taxonomic status of these suggested lineages.

Genetic diversity was estimated for the different lineages/haplogroups as identified in the phylogenetic analyses and network. Diversity indices such as haplotype diversity [92] and nucleotide diversity, π [93] were calculated for the whole sample and individual lineages/haplogroups excluding missing data using DnaSP, version 4.10.9 [94].

All phylogenetic and dating analyses were based on the 137 identified alleles representative of the overall diversity. Outgroup selection for phylogenetic analyses within *M. namaquensis *was problematic due to several proposed hypotheses of evolutionary relationships between *Micaelamys *and other murids [[3,95] and references therein]. In a preliminary analysis *A. chrysophilus*, *A. ineptus*, *Parotomys brantsi*, *Dasymys incomtus*, *Rattus rattus*, *M. musculus*, *Rhabdomys pumilio *and *Arvicanthis somalicus *were included as outgroups. These species are distant to the ingroup but given their sister group relationship (results not shown) and former congeneric status [33], the two *Aethomys *species were selected as outgroups in further analyses. However, *R. rattus *and *M. musculus *were used in the BEAST analysis since a fossil calibration point was available (see section on molecular clocks).

A likelihood ratio test as implemented in Modeltest, version 3.06 [96] was used to determine the best-fit model of DNA substitution for the 631 bp cyt *b *sequences under the Akaike Information Criterion (AIC) and to estimate the relevant parameters under this model. The best-fit General-Time-Reversible (GTR) model of substitution with a gamma correction (Γ = 1.09; [97]), and a proportion of invariable sites (I = 0.54) was applied in phylogenetic analyses. The Bayesian analysis was conducted using MrBAYES version, 3.1.2 [98]. Four chains were run for 5 × 10^6 ^generations using random starting trees and flat priors. Trees and parameters were recorded every 100^th ^generation. Two runs were performed simultaneously and split frequencies were compared every 100^th ^generation to ensure convergence of the runs. Both runs used the default heating and swap parameters. The first 5 000 generations (10%) were excluded as the burn-in. A 10% burn-in was sufficient to ensure that trees were only sampled from the region of stationarity.

To estimate a rate of evolution and dates of divergence between these lineages, a log-normal relaxed-clock analysis was performed using the speciation model: Yule process as implemented in BEAST, version 1.4.7 [99]: three independent runs of 20 × 10^6 ^generations each were performed. A specific rate of change calibrated for murid rodents was determined since murid mtDNA has been shown to evolve at a faster rate than other rodents [100]. As a calibration point, sequence data from *R. rattus *and *M. musculus*, with a divergence date estimated at 12 Mya based on fossil records, was used [101]. In contrast, other divergence dates (between 10 and 41 Mya) have been suggested for *Rattus *and *Mus *[102-104]. The divergence date of 12 Mya was followed since it is based on the fossil record and provided a rodent-specific calibration. The use of a non-rodent calibration point results in divergence times that are much older than the paleontological record [102].

Posterior distributions of parameters were approximated by Markov Chain Monte Carlo (MCMC) [105] sampling [99] with samples drawn every 1 000^th ^iteration over a total of 20 × 10^6 ^generations, excluding the first 4 000 generations as the burn-in. Three independent analyses were run and the results were combined using LogCombiner, version 1.4.7 [99]. Independent runs were combined in Tracer, version 1.4 [106], which provide options for examining effective sample size (ESS) values and to evaluate adequate mixing and convergence to the stationary distribution. This was evaluated by the Bayesian skyline plot [107] as calculated in Tracer, version 1.4 [106]. Posterior estimates for rate and divergence date estimates were similar between runs. The final tree created from the three independent runs was viewed in FigTree, version 1.2.2 [99]. The International Commission on Stratigraphy (ICS) published a timescale chart in 2009. This chart was used in order to interpret divergence dates as observed in the BEAST analysis (see: strategra http://www.stratigraphy.org).

The minimum number of mutational steps between *M. namaquensis *alleles was determined from a distance matrix using MINSPNET [108] and TCS [109]. Frequencies and geographic distributions of different alleles were used to depict geographical and potential ancestor-descendant relationships among identified allele sequences. To illustrate contrasting patterns of intra-clade structuring, TCS and MINSPNET were also used to produce minimum-spanning networks for all divergent haplogroups identified.

A Mantel test as implemented in Mantel Nonparametric Test Calculator, version 2.0 [110] was used to test for isolation by distance within identified lineages/haplogroups. The test uses a permutation procedure (1 000 permutations) to determine the significance of the correlation between genetic versus geographic distances. Some of the groups could not be subjected to the Mantel test, as too few individuals were sampled.

For purposes of the present study, we follow the biome/bioregion definitions as defined by [45], although biome definitions are largely identical irrespective of the authority. See Figures 1 and 5 for biome information and [45] for more information on biomes/bioregions. Maximum likelihood (ML) [111,112] was used to test for the association between lineages and the major biomes of southern Africa. ML as implemented in PhyML, version 3.0 [113,114] was conducted using 100 random addition replicates and was based on a heuristic search using the tree bisection-reconnection (TBR) option; the best fit substitution model parameters were applied. Support values for internal nodes were determined using bootstrap analysis [115] with 100 iterations performed on a computer cluster.

## List of abbreviations

AIC: Akaike Information Criterion; CYT *B*: cytochrome *b*; ESS: effective sample size; GTR: Gerneral-Time-Reversible; KYA: thousand years ago; LGM: last glacial maximum; MTDNA: mitochondrial DNA; MCMC: Monte Carlo Markov Chain; ML: Maximum likelihood; MYA: million years ago; PCR: Polymerase chain reaction; TMRCA: time to the most common recent ancestor.

## Authors' contributions

PB and CTC initiated the study and obtained research funding. All authors conceptualised the study. IMR collected specimens and was responsible for generating and interpreting the data. All authors contributed to the writing and improvement of the manuscript. All authors read and approved the manuscript.

## Supplementary Material

Additional file 1 **Variable sites of 137 mtDNA cyt *b *alleles of *Micaelamys namaquensis***. Variable sites of 137 mitochondrial DNA (mtDNA) cytochrome *b *(cyt *b*) alleles (631 base pairs) of *Micaelamys namaquensis *from southern Africa. Variable positions one and 631 correspond to positions 14139 and 14770 of *Mus musculus *[86]. Dots (.) indicate identity to the base in the references sequence NH001. Haplotype order corresponds to the different haplogroups/lineages that were identified in the phylogenetic/phylogeographic analyses. Click here for file

Additional file 2 **Frequencies and localities of 137 mtDNA cyt *b *alleles of *Micaelamys namaquensis***. Frequencies and localities of 137 mitochondrial DNA (mtDNA) cytochrome *b *(cyt *b*) alleles of *Micaelamys namaquensis *from southern Africa. Numbers in parentheses represent locality numbers and the number of individuals examined per locality. Haplotype order corresponds to the different haplogroups/lineages that were identified in the phylogenetic/phylogeographic analyses. Geographic coordinates of localities are indicated in Additional file 5. Click here for file

Additional file 3 **Support for the different lineages using different methods**. Support for the different lineages using different methods. "*" indicates resolution and statistical support, "**+**" indicates resolution but no statistical support, and "-" indicates unresolved. Click here for file

Additional file 4 **Mantel test for the different haplogroups/lineages within *Micaelamys namaquensis***. Mantel test results (mitochondrial DNA (mtDNA) cytochrome *b *(cyt *b*)) for the different haplogroups/lineages within *Micaelamys namaquensis *from southern Africa as defined by an allele network and phylogenetic analyses. In most analyses, the standard normal variate (*g*) was smaller than the critical value of 2.575. See Figures 2, 3, 4 and 5 for the genetic and geographic distinction of the lineages. Mantel test for some lineages were not shown as a result of small sample sizes. *P*-values in bold indicate significant correlation between geographic and genetic distance. Click here for file

Additional file 5 **Geographic coordinates of all collecting localities of *Micaelamys namaquensis***. Geographic coordinates of all collecting localities of *Micaelamys namaquensis *from southern Africa analysed in the present study. Numbers 1 - 95 correspond to those in Additional file 6. Click here for file

Additional file 6 **Collecting localities of samples of *Micaelamys namaquensis***. Collecting localities of samples of *Micaelamys namaquensis *from southern Africa. Numbers correspond to the locality numbers and names in Additional file 5. Click here for file

Additional file 7 **Permits and permit numbers**. Permits and permit numbers for the nine provinces representing South Africa and permits for Botswana, Swaziland and Namibia. Click here for file
