# Supplementary material for: Bioregion heterogeneity correlates with extensive mitochondrial DNA diversity in the Namaqua rock mouse, Micaelamys namaquensis (Rodentia: Muridae) from southern Africa - evidence for a species complex
Source: BMC Evol Biol. 2010 Oct 13;10:307. doi: 10.1186/1471-2148-10-307 (PMC2967545; doi:10.1186/1471-2148-10-307)
Supplement: Additional file 1 — Variable sites of 137 mtDNA cyt b alleles of Micaelamys namaquensis . Variable sites of 137 mitochondrial DNA (mtDNA) cytochrome b (cyt b) alleles (631 base pairs) of Micaelamys namaquensis from southern Africa. Variable positions one and 631 correspond to positions 14139 and 14770 of Mus musculus [86]. Dots (.) indicate identity to the base in the references sequence NH001. Haplotype order corresponds to the different haplogroups/lineages that were identified in the phylogenetic/phylogeographic analyses. [file 1471-2148-10-307-S1.DOC]

Haplotype Variable positions within the 631 bp fragment of cytochrome *b* gene

Number

111111111111111111112222222222222222222223333333333333333333333333333444444444444444444444444455555555555555555555555555555555566666666666660

11111111222222333444556667778899000112223334445667780011112333334457888990000122223444455566777888999012222333455556677777889900112233344445556666677888889999900001111112233

58901234589012347038258460372584739258151572561459584891704692124573686258171369813789258914735589147069570369468836795812358362814692347803692381347836024581247803694567890101

Lineage H

NH001 CACATCCGAAACCCATTACTTCCTTCCGACCAAACTATTATTGATTTCTACCCCCTCCGCACACAGATAACATACAGTACACCTCACCAGGCTTCCCGATATACAATAAATACCGCACCTAACATAATAACATCCCTGACCCTTACTCTATCGTTTTACACCGCAACCAAC?????

NH002 ??????????????????....................................................................................G.......................................................T............AACAC

NH003 ????????......................................................................................................................................................T............AAC??

NH004 ??????????......C....T...T................A...................................................................................................................T.????????????????

NH005 ................C....T.................................C......G.....................T.....A...............C.....T.T........................................C.CT...A........AAC??

NH006 ................C....T.................................C......G.....................T.....A...............C.......T.....................C....................CT...A........?????

NH007 ................C....T.....A............................................................................................................................A.....T.????????????????

NH008 ................C....T....................................................................A..........C........................................................T..........CAAC???

NH009 ................C....T....................................................................A.....................................G.............................T............AACAC

NH010 ???????????????.C....T.....A.......C......................................................A...................................................................T.......??????????

NH011 ................C....T.....A.........................................................................C........................................................T...??????????????

NH012 ................C....T....................................................................A..........C........................................................T............AAC??

NH013 ................C....T.................G......................................................................................................................T.......??????????

Lineage D

NH014 ................C....T.........G..T..C....A............C.T..TT.TG............CG.....T.....AT.............T......T.T.....G......CG.......C......C......C.AC...C.............AACCC

NH015 ................C....T.....A...G..T..C....A............C.T..TT.TG............CG.....T.....AT.............T......T.T.....G......CG......TC......C......C.AC...C.............AAC??

NH016 ................C....T.....A...G..T..C....A............C.T..TT.TG............CG.....T.....AT.............T......T.T.....G......CG.......C......C......C.AC...C.............AACCC

NH017 .........G......C....T.........G..T..C....A............C.T..TT.TG............CG.....T.....AT.............T......T.T.....G......CG.......C......C......C.AC...C.............AACCC

NH018 ................C....T.........G..T..C....A............C.T..TT.TG............CG.....T.....AT.............T......T.T.....G......CG.......C......C......C.AC.C.C.............AACCC

NH019 ................C....T.........G..T..C....A............C.T..TT.TG............CG.....T.....AT.............T......T.T.....G......C.......TC......C......C.AC...C.............AACCC

NH020 ................C....T.........G..T..C....A............C.T..TT.TG............CG.....T.....AT...........T.T......T.T.....G......CG.......C......C......C.AC...C.............AACCC

NH021 ................C....T.........G..T..C....A............C.T..TT.TC............CG.....T.....AT.............T......T.T.....G......CG.......C......C......C.AC...C.............AACCC

NH022 ?????????????...C....T.........G..T..C....A............C.T..TT.TG............CG.....T.....AT.............T......T.T.....G......CG......TC......C......C.AC...C..????????????????

NH023 ................C....T.........G..T..C....A............C.T..TT.TG............CG.....T.....AT.......C.....T......T.T.....G......CG.......C......C......C.AC...C.............AAC??

NH024 ................C....T.........G..T..C....A............C.T..TT.TG............CG.....T.....AT.............T......T.T.....G......CG.....T.C......C......C.AC...C.......C??????????

NH025 ................C....T.........G..T..C....A............C.T..TT.TG............CG.....T.....AT.............T......T.T.....G......CG.....T.C......C......C.AC...C.............AACCC

NH026 ................C....T.........G..T..C....A............C.T..TT.TG............CG.....T.....AT.............T......T.TT....G......CG.......C......C......C.AC...C.............AACCC

NH027 ................C....T.........G..T..C....A............C.T..TT.TG............CG.....T.....AT.............T......T.T.....G......CG.......C.....CC......C.AC...C.............AACCC

NH028 ??????????......C....T.........G..T..C....A............C.T..TT.TG............CG.....T.....AT.............T......T.T.....G......CG..............C......C.AC...C.....T.......AAC??

NH029 ?...............C....T.........G..T..C....A............C.T..TT.TG.....T......CG.....T.....AT.............T......T.T.....G......CG.......C......C......C.AC...CT............AACCC

NH030 ................C....T.........G..T..C....A............C.T..TT.TG............CG.....T.....AT.............T......T.T.....G......CG.......C......C......C.AC...C........T.C.ACAA??

NH031 ??????????......C....T.........G..T..C....A............C.T..TT.TC............CG.....T.....AT.............T......T.T.....G......CG.......C......C......C.AC...C........T.C.ACACCC

NH032 ................C....T.........G..T..C....A............C.T..TT.T.............CG.....T.....AT.............T......T.T.....G......CG......TC......C......C.AC...C.............AACCC

NH033 T....A..........C....T.........G..T.GC....A............C.T..TT.TG............CG.....T.....AT.............T......T.T.....G......CT......TC......C......C.ACCC.C....??????????????

NH034 T...............C....T.........G..T..C....A.....C......C.T..TT.TG............CG.....T.....AT.............T......T.T.....G......CT......TC......C......C.AC.C.C.............AAC??

NH035 T...............C....T.........G..T..C....A............C.T..TT.TG............CG.....T.....AT.............T......T.T.....G......CT......TC......C......C.AC.C.C.............AACCC

NH036 ?????...........C....T.........G..T..C....A............C.T..TT.TG............CG.....T.....AT.............T......T.T.....GG.....CT......TC......C......C.AC.C.C.............AAC??

NH037 .......T........C....T.........G..T..C....A..C.........C.T..TT.TG............CG.....T.....AT.............T......T.T.....G...C..CG......TC......C......C.AC...C.............AACCC

NH038 ??????..........C....T.........G..T..C....A............C.T..TT.TG............CG.....T.....AT.............T......T.T.....G......CT......TC......C......C.AC.C.C...G??????????????

NH039 ?????A.CG.......C....T.........G..T..C....A............C.T..TT.TG............CG.....T.....AT.............T......T.T.....G......CT......TC......C......C.AC.C.C.............AACCC

NH040 ????????........C....T.....A...G..T..C....A............C.T..TT.TG............CG.....T.....AT.............T......T.T.....G......C.......TC......C......C.AC...C......????????????

NH041 ................C....T.....A...G..T..C....A............C.T..TT.TG..C.........CG.....T.....AT.............T......T.T.....G......CG......TC...T..C......C.AC...C.............AAC??

NH042 ???????????.....C....T.........G..T..C....A............C.T..TT.TG..C.........CG.....T.....AT.............T......T.T.....G......CG......TC...T..C......C.AC...C........??????????

NH043 ................C....T.........G..T..C....A............C.T..TT.TG..C.........CG.....T.....AT.............T......T.T............CG......TC...T..C......C.AC...C.............A????

NH044 ................C....T.........G..T..C....A............C.T..TT.TGA...........CG.....T.....AT.............T......T.T.....G......C.......TC......C......C.AC...C.............AACCC

Lineage A2

NH045 ...............CC......C..........T.............C........T..TT.T.....G........G.....T.....AT...T................T.C....C.......C....C...C..T.T........C.A....CT............AACAC

NH046 ...............CC......C..........T......................T..TT.T.....G........G.....T.....AT...T................T.C....C.......C....C...C..T.T........C.A....CT............AAC??

NH047 ...............CC......C..........T.............C........T..TT.T.....G........G.....T..T..AT...T................T.C....C.......C....C...CA.T.T........C.A....CT........?????????

NH048 ................C......C......T...T......................T..TT.T...C.G........G.....T.....AT...T................T.C....C.......C....C...C..T..........C.A....CT............AAC??

NH049 ...............CC......C..........T......................T..TT.T....GG........G.....T.....AT...T................T.C....C.......C....C...C..T.TC.......C.A....CT...........??????

NH050 ?????????????????...C..C..........TC.....................T..TT.T.....G........G.....T.T.G.AT...T................T.C...TC.......CG...C...C..T.T........C.A....CT.????????????????

NH051 ...............CC...C..C..........TC.....................T..TT.T.....G........G.....T.T.G.AT...T............G...T.C....C.......CG...C...C..T.T........C.A....CT............AACAC

NH052 ...............CC...C..C..........TC............C........T..TT.T.....G........G.....T.T.G.AT...T............G...T.C....C.......CG...C...C..T.T........C.A....CT............AACAC

NH053 ................C......C..........T......................T..TT.T...C.G........G.....T...G.AT...T....G...........T.C....C.......C....C...C..T..........C.A....CT............AAC??

NH054 ????????????????????...C..........T......................T..TT.T...C.G........G.....T...G.AT...T................T.C....C.......C....C...C..T.......T..C.????????????????????????

NH055 ................C....T.C..........T......................T..TT.T...C.G........G.....T...G.AT...T................T.C....C.......C....C...C..T..........??????????????????????????

NH056 ...............CC......C..........TC.............G.......T..TT.T.....G..............T...G.AT...T..G.............T.T....C.....???????????????????????????????????????????????????

NH057 ??????????????????.....C..........T..............G.......T..TT.T.....G..............T...G.AT...T..G.............T.T....C.......C....CT..C..T....G.....C.A....C??????????????????

NH058 ...............CC......C..........T..............G.......T..TT.T.....G..............T...G.AT...T..G.............T.T....C.......C....CT..C..T....G..T..C.A....CT......???????????

NH059 ???????????????????????????????????...C..........G.......T..TT.T.....G..............T...G.AT...T..G.............T.T....C.......C....CT..C..T....G.....C.A....CT............AACAC

NH060 ...............CC......C..........T..............G.......T..TT.T....GG..............T...G.AT...T..G.............T.T....C...G...C....CT..C..T....G.....C.A....CT......???????????

NH061 .....A......T...C......C..........T.........C....G.......T..TT.T..G..G.......CG.....T...G.AT...T.....C..........T.T....C.......C....C...C..T..........C.A....CT?????????????????

NH062 ?????????????????......CC.........T..............G.......T..TT.T..G..G........G.....T...G.AT...T.....C..........T.T....C.......C....C...C..T..........C.A....CT............AAC??

NH063 ????????????????C......C..........T.........C....G.......T..TT.T..G..G........G.....T...G.AT...T.....C..........T.T....C.......C....C...C..T..........C.A....CT.......??????????

NH064 ................C......C..........T...............T......T..TT.T.....G..C.....GT....T...G.AT...T................T.C....C.......C....C...C..T..........C.A....CT......???????????

NH065 ?????????.......C......C..........T...............T......T..TT.T.....G......A.G.....T...G.AT...T................T.C....C.......C....C...C..T..........C.A....CT............AAC??

NH066 ................C......C..........T......................T..TT.T.A.C.G........G.....T...G.AT.C.T................T.C....C.......C....C...C..T..........C.A....CTG????????????????

NH067 ................C......C..........T...........C..........T..TT.T.A.C..........G.....T...G.AT.C.T................T.C....C.......CG...C...C..T..........C.A....CT............AAC??

NH068 ??????????......C......C..........T..............G.......T..TT....G..G........G.....T...G.AT...T.....C..........T.T....C.......C....C...C..T..........C.A....CT.......??????????

NH069 .....A......T...C......C..........T..............G.......T..TT.T..G..G........G.....T...G.AT...T.....C......G...T.T....C.......C....C...C..T..........C.A....CT............AAC??

NH070 .....A......T...C......C..........T..............G.......T..TT.T..G..GT.......G.....T...G.AT...T.....CG.........T.T....C.......C....C...C..T..........C.A....CT..???????????????

NH071 .....A......T..CC......C..........T..............G.......T..TT.T..G..G........G.....T...GAAT...T.....C........ACT.T....C.......C....C...C..T..C.......C.A....CT......???????????

NH072 ..T............CC......C..........TC.....................T..TT.T.....G........G.....T.....AT...T................T.C....C.......C....C...C..T.T........C.A....CT.....T......AAC??

Lineage B3

NH073 ?????????????????....T............................T...T..T..TT.T...........G........T..T..AT...T......C.....G...TTT...........G...T.C...C..T..........C.A...CCT.......??????????

NH074 ?????????????????????????.........................T...T..T..TT.............G........T..T..AT...T......G.....G...TTT...............T.C...C..T..........C.A...CCT......???????????

NH075 ?CATC.GA..C..ATCC....T.....A............C.........T...T..T..T..T...C................T..TG.AT...T............G...TTC...............T.C...C..T..........C.A...CCT.......??????????

NH076 T...............C....T............................T...T..T..T..T...C................T..TG.AT...T............G...TTC...............T.C...C..T..........C.A...CCT.????????????????

NH077 T...............C....T............................T...T..T..TT.T...........G........T..T..AT...T......G.....G...TTT...............T.C...C..T..........C.A...CCT.......??????????

NH078 T...............C....T............................T...T..T..TT.T...........G.A......T..T..AT...T......G.....G...TTT...........G...T.C...C..T..........C.A...CCT..........???????

NH079 T...............C....T............................T...T..T..TT.T...........G........T..T..AT...T......G.....G...TTT...........G...T.C...C..T..........C.A...CCT............AAC??

NH080 T...............C....T............................T...T..T..TT.T...........G........T..T..AT...T......G.....G...TTT...............T.C...C..T..........C.A..CCCT............AAC??

NH081 ???????.........C....T............................T...T..T..TT.T...........G........T..T..AT...T......G.....G...TTT..........G....T.C...C..T..........C.A...CCT.....????????????

NH082 ????????????????????.T............................T...T..T..TT.T...........G........T..T..AT...T......C.........TTT...........G...T.C...C..T..........C.A...CCT............AAC??

NH083 T...............C....T............................T...T.....TT.T...........G........T..T..AT...T......G.....G...TTT...............T.C...C..T..........C.A...CCT.....????????????

Lineage B2

NH084 ................CG...T............................T.T..C....TT.T..............G..T..T..TG.AT...T.............T..TTT............CG...C...C..T...C......C.A...CCT............AAC??

NH085 ................CG...T..........G..C..............T.T..C.T..TT.T.................T..T..TG.AT...T.............T..TTT............C....C...C..T...C......C.A...CCT............AACAC

NH086 ................CG...T..........G..C..............T.T..C.T..TT.T.................T..T..TG.AT...TG............T..TTT............C....C...C..T...C......C.A...CCT............AAC??

Lineage F

NH087 ................C..A.T...TT....................T....T....T..T..TG...................T..T..AT...T.A..............T.T..........G.C........C..T..........C.A....CT.T......T...AACAC

NH088 ................C....T...TT....................T....T....T..T..TG.........T.........T..T..AT...T................T.T..........G.C........C..T..........C.A....CT........?????????

Lineage A5

NH089 ??????????????????????????????............A..............T..TT.TG........G....G....CT.....AT...T................T.T.G......G...C....C...C..?????????????????????????????????????

NH090 ................C......C.....T.....C......A.......T.........CT.T.........G.......T.CT.....AT...T................T.T.G......G...C....C...C..T..........C.A...CCT............?????

NH091 ????????????????C......C.....T.....C......A.......T.........CT.T.........G....G..TTCT.....AT...T................T.T.G......G...C....C...C..T..........C.A...CCT......???????????

Lineage A4

NH092 ????????????????C....T.C..T............................C.T..TT.TG...................T...G.AT...T............G...T.T..T.........C....C...C.TT..........C.A...CCT....?????????????

NH093 ................C....T.C..T.G..........................C.T..TT.TG...................T...G.AT...T................T.T..T.........C....C...C..T..........C?????????????????????????

NH094 ................C....T.C..T............................C.T..TT.TG...................T...G.AT...T............G...T.T..T.........C....C...C..T..........C.A...CCT............AACAC

NH095 T...............C......C..T..............C...............T..TT.TG...................T.....AT...T............G...T.T..T.........C....C...C..T..........C.A...CCT.......??????????

NH096 ?????????.......C......C..T..............................T..TT.TG...................T...G.AT..TT................T.T..T.........C....C...C..T..........C.A...CCT.......??????????

Lineage C

NH097 ................C....T.........G...C..........C..........T..TT.T.....G.......CG.G...T.TT..AT...T................T.T.......G....CG.......C..T...C......C.A..CCCT............AACAC

NH098 ?...............C....T.....A...G..TC..........C..........T..TT.T.....G.......CG.....T.TTG.AT...T........G.......T.T.......G....C????????????????????????????????????????????????

NH099 ?????????.......C....T.........G...C..........C..........T..TT.T.....G.......C..G...T.TT..AT...T........G.......T.T...T...G....CG.......C..T...C......C.A..CCCT.......??????????

NH100 ................C....T.........G...C..........C..........T..TT.T.............CG.G...T.TT..AT...T........G...G...T.T.......G....CG.......C..T...C......C.A..CCCT............AACAC

NH101 ................C....T.C.......G...A..........C..........TA.TT.T.A...........CG.G...T.TT..AT...T........G.......T.T.......G....CG.......C..T...C......C.A..CCCT......???????????

NH102 ................C....T.........G...C..........C..........T..TT.T.............CG.G...T.TT..AT...T........G...G...T.T.......G....CGG......C..T...C......C.A..CCCT............AACAC

NH103 ???????????.....C....T.........G...C..........C..........T..TT.T.....G.......CG.....T.TTG.AT...T........G.......T.T.......G....CG.......C..T..........C.A....CT......???????????

NH104 ................C....T.....A...G...C......C...C..........T..TT.T.....G.......CG.....T.TTG.AT...T........G.......T.T.......G....CG.......C..T...C......C.A..C.CT............AACAC

NH105 ................C....T.....A...G...C.......G..C..........T..TT.T.....G.......CG.....T.TTG.AT...T........G.......T.T.......G....CG.......C..T...C......C.A..C.CT............AAC??

NH106 ................C....T.C.......G...A..........C..........T..T..T.A...........CG.G...T.TT..AT...T.....C..G.......T.T.......G....CG.......C..T...C......C.A..CC???????????????????

NH107 ................C....T.........G...C..........C..........T..TT.T.............CG.G...T.TT..AT...T........G...G...T.T.......G....CG.......C..T...C......C.A..CCCT............AACAT

Lineage G

NH108 T...............C....TT.......................C..........T.TT..TG.............G.....T.....AT....................T.T...T........CG..G....C.G...........C......CT.T.A........AACAC

NH109 T...............C....TT.......................C..........T.TT..TGA............G.....T.....AT...............G....T.T...T........CG..G....C.G...........C.A....CT.T.A........AACAC

NH110 T...............C....TT.......................C..........T.TT..TG.............G.....T.....AT...............G....T.T...T........CG..G....C.G...........C......CT.T..........AACAT

NH111 T...............C....TT.......................C..........T.TT..T..............G.....T.....AT...............G....T.T...T........CG..G......G...........C......CT.T..........AACAC

NH112 T...............C.A..TT.......................C.........TT.TT..T..............G.....T.....AT...............G....T.T...T........CG..G....C.G...........C......CT.T..........AACAC

NH113 T...............C....TT..........G............C..........T.TT..TG.............G.....T.....AT...............G....T.T...T........CG..G....C.G...........C......CT.T..........AACAC

NH114 T...............C....TT..........G............C..........T.TT..TG.............G.....T.....AT...............G....T.T...T........CG..G....C.G...........C.A....CT.T..........A????

NH115 T...............C....TT.......................C..........T.TT..TG...G.........G.....T......T...............G....T.T...T........CG..G....C.............C......CT.T..........AACAC

NH116 T...............C....TT.......................C..........T.TT..TG...G.........G.....T......T..............CG....T.T...T........CG..G....C.............C......CT.T..........AACAC

NH117 ???????????????CC....TT.......................C..........T.TT..TG...G.........G.....T......T...............G....T.T...T........CG..G....C.............C......CT.T..........AACAC

NH118 T...............C....TT.......................C..........T.TT..TG...G.........G..T..T......T...............G....T.T...T........CG..G....C.............C......CT.T..........AACAC

NH119 T...............C....TT.......................C..........T.TT..TG.............G.....T.....AT...............G....T.T...T........CG..G....C.G...........C......CT.T..........AACAC

Lineage E

NH120 T...............C..........A...G......C...A..........T...T..TT..G.............GT...C......AT...T................T.T............C........C..T.....T.TC.C.AC.C.CT............AAC??

NH121 T...............C..........A...G..........A..............T..TT........T........T...C......AT...T................T.T.........C..CG.......C..T.....T.T..C.AC.C.CT............AACAC

NH122 ?????????????...C..........A...G..........A..............T..TT..G.....T.......GT...C....G.AT...T...C............T.T.........C..CG.......C..T.....T.T..C.AC.C.CT..........???????

NH123 T...............C..............G..........A..............T..TT........T........T...C......AT...T................T.T.........C..CG.......C..T.....T.T..C.AC.C.CT............AACAC

NH124 ??????????????????????????.....G......C...A........A.T...T..TT..G.............GT...C......AT...T................T.T............C........C..T......GT.GA?????????????????????????

NH125 T...............C..............G..........A..............T..TT........T........T...C....G.AT...T................T.T.........C..CG.......C..T.....T.T..C.AC.C.CT............AACAC

NH126 T..........T....C..............G..........A..............T..TT........T........T...C......AT...T................T.T.........C..CG.......C..T.....T.T..C.AC.C.CT............AACAC

NH127 T...............C..............G..........A..............T..TT........T........T..TC....G.AT...T................T.T.........C..CG.......C..T.....T.T..C.AC.C.CT.....????????????

NH128 ??????..........C..............G..........A..............T..TT..G.....T.......GT...C....G.AT...T................T.T.........C..CG.......C..T.....T.T..C.AC.C.CT............AACAC

NH129 T...............C..............G..........A..............T..TT........T........T.TTC....G.AT...T................T.T.........C..CG.......C..T.....T.T..C.AC.C.CT............AACAC

NH130 ????????????....C..............G...C.C...CA..............T..TT..G......GC.....GT...C....G.AT....................T.T............CG.......C..T.....T.T..C.AC.C.C??????????????????

Lineage A1

NH131 ???????????????????????????????....C.....................T..TT.TG.............G.....T.....AT...T................T.T............C....C..?????????????????????????????????????????

Lineage A3

NH132 ??????..........C........................................T..TT.TG...................T...G.ATC..T................T.TA...........C....C...C.............CTA...CC??????????????????

NH133 ................C......C................................TT..TT.TG...G...............T...G.AT...T............G...T.TA...........C....C...C..T..........C.A...CCT..T....??????????

NH134 ................C......C...A.............................T..TT.T....................TG..G.AT...T................T.TA...........C....C...C..T..........C.A...CCT..T??????????????

NH135 ??..............C......C.................................T..TT.TG...................T...G.AT...T................T.TA...........C....C...C..T..........C.A...CC...T.........AAC??

NH136 ??????????????????.....C.................................T..TT.TG...................T...G.AT...T................T.TA...........C....C...C..T..........C.A...CCT..T.........AACAC

Lineage B1

NH137 ????????????....CG...T............................T....C....TT.T..............G.....T.....AT....................T.T............CG...C...C..T...C......C.A...????????????????????
