# Supplementary material for: Bioregion heterogeneity correlates with extensive mitochondrial DNA diversity in the Namaqua rock mouse, Micaelamys namaquensis (Rodentia: Muridae) from southern Africa - evidence for a species complex
Source: BMC Evol Biol. 2010 Oct 13;10:307. doi: 10.1186/1471-2148-10-307 (PMC2967545; doi:10.1186/1471-2148-10-307)
Supplement: Additional file 2 — Frequencies and localities of 137 mtDNA cyt b alleles of Micaelamys namaquensis . Frequencies and localities of 137 mitochondrial DNA (mtDNA) cytochrome b (cyt b) alleles of Micaelamys namaquensis from southern Africa. Numbers in parentheses represent locality numbers and the number of individuals examined per locality. Haplotype order corresponds to the different haplogroups/lineages that were identified in the phylogenetic/phylogeographic analyses. Geographic coordinates of localities are indicated in Additional file 5. [file 1471-2148-10-307-S2.DOC]

| Haplotype number | Frequency of haplotype | Localities (Locality number as indicated in Figure 3 and Additional file 5, number of individuals collected) |
| --- | --- | --- |
| **Lineage H** |  | **17** |
| NH001 | 1 | Baltimore (7, 1) |
| NH002 | 3 | Botswana: Terrafou (3, 2), Alldays (5, 1) |
| NH003 | 1 | Blouberg Nature Reserve (6, 1) |
| NH004 | 1 | Botswana: Terrafou (3, 1) |
| NH005 | 1 | Botswana: Elephant Sands (1, 1) |
| NH006 | 1 | Botswana: Francistown (2, 1) |
| NH007 | 1 | Musina Nature Reserve (4, 1) |
| NH008 | 1 | Musina Nature Reserve (4, 1) |
| NH009 | 1 | Botswana: Terrafou (3, 1) |
| NH010 | 2 | Botswana: Terrafou (3, 2) |
| NH011 | 1 | Botswana: Francistown (2, 1) |
| NH012 | 1 | Musina Nature Reserve (4, 1) |
| NH013 | 2 | Musina Nature Reserve (4, 2) |
| **Lineage D** |  | **118** |
| NH014 | 24 | Botswana: Kasane (8, 1), Upington (9, 10), Augrabies (15, 9), Gariep Nature Reserve (21, 1), Kirkwood (28, 1), Kakamas (89, 2) |
| NH015 | 1 | Springbok (18, 1) |
| NH016 | 3 | Upington (9, 3) |
| NH017 | 3 | Upington (9, 2), Augrabies (15, 1) |
| NH018 | 4 | Upington (9, 4) |
| NH019 | 7 | Botswana: Kasane (8, 3), Hoopstad (10, 2), Kimberley (13, 1), Bloemfontein (16, 1) |
| NH020 | 1 | Pofadder (17, 1) |
| NH021 | 3 | Loxton (24, 3) |
| NH022 | 1 | Springbok (18, 1) |
| NH023 | 1 | Hopetown (19, 1) |
| NH024 | 1 | Upinton (9, 1) |
| NH025 | 9 | Upington (9, 9) |
| NH026 | 1 | Lady Grey (22, 1) |
| NH027 | 18 | Upington (9, 18) |
| NH028 | 1 | Hopetown (19, 1) |
| NH029 | 2 | Augrabies (15, 2) |
| NH030 | 11 | Botswana: Kasane (8, 1), Upington (9, 4), Augrabies (15, 1), Gariep Nature Reserve (21, 3), Karoo National Park (25, 2) |
| NH031 | 1 | Loxton (24, 1) |
| NH032 | 2 | Brandfort (14, 1), Bloemfontein (16, 1) |
| NH033 | 1 | Oudtshoorn (27, 1) |
| NH034 | 3 | Kirkwood (28, 2), Porterville (50, 1) |
| NH035 | 9 | Gariep Nature Reserve (21, 1), Lady Grey (22, 1), Karoo National Park (25, 1), Matjiesfontein (26, 2), Oudtshoorn (27, 4) |
| NH036 | 1 | Jamestown (23, 1) |
| NH037 | 1 | Willem Pretorius Nature Reserve (11, 1) |
| NH038 | 1 | Oudtshoorn (27, 1) |
| NH039 | 1 | Kirkwood (28, 1) |
| NH040 | 1 | Wepener (20, 1) |
| NH041 | 1 | Pofadder (17, 1) |
| NH042 | 1 | Springbok (18, 1) |
| NH043 | 3 | Springbok (18, 3) |
| NH044 | 1 | Boshof (12, 1) |
| **Lineage A2** |  | **41** |
| NH045 | 3 | Boshoek (38, 1), Kgaswane Mountain Reserve (39, 1), Ezemvelo Nature Reserve (41, 1) |
| NH046 | 3 | Lapalala Nature Reserve (31, 2), Rooibokkraal (32, 1) |
| NH047 | 1 | Boshoek (38, 1) |
| NH048 | 2 | Lajuma Mountain Retreat (29, 1), Schweizer-Reneke (67, 1) |
| NH049 | 1 | Ellisras (30, 1) |
| NH050 | 1 | Brits (40, 1) |
| NH051 | 2 | Kgaswane Mountian Reserve (39, 1), Ventersdorp (44, 1) |
| NH052 | 2 | Kgaswane Mountain Reserve (39, 2) |
| NH053 | 1 | Lapalala Nature Reserve (31, 1) |
| NH054 | 1 | Hoedspruit (37, 1) |
| NH055 | 1 | Hoedspruit (37, 1) |
| NH056 | 1 | Machadodorp (93, 1) |
| NH057 | 1 | Ezemvelo Nature Reserve (41, 1) |
| NH058 | 3 | Josefsdal Nature Reserve (46, 1), Amsterdam (47, 2) |
| NH059 | 1 | Kruisrivier Nature Reserve (42, 1) |
| NH060 | 1 | Kruisrivier Nature Reserve (42, 1) |
| NH061 | 1 | Potchefstroom (43, 1) |
| NH062 | 1 | Brits (40, 1) |
| NH063 | 1 | Brits (40, 1) |
| NH064 | 1 | Bela-Bela (36, 1) |
| NH065 | 1 | Brits (40, 1) |
| NH066 | 1 | Musina Nature Reserve (4, 1) |
| NH067 | 1 | Lajuma Mountain Retreat (29, 1) |
| NH068 | 2 | Ben Alberts Nature Reserve (33, 1), Thabazimbi: Waterval (34, 1) |
| NH069 | 3 | Vredefort (45, 3) |
| NH070 | 1 | Ezemvelo Nature Reserve (41, 1) |
| NH071 | 1 | Vredefort (45, 1) |
| NH072 | 2 | Botswana: Gaborone (35, 2) |
| **Lineage B3** |  | **17** |
| NH073 | 2 | Porterville (50, 2) |
| NH074 | 1 | Robertson (52, 1) |
| NH075 | 2 | Cederberg: Jamaka (49, 2) |
| NH076 | 1 | Cederberg: Boscherberg (48, 1) |
| NH077 | 3 | Jonaskop (51, 2), Die Galg (54, 1) |
| NH078 | 2 | Porterville (50, 2) |
| NH079 | 1 | Cederberg: Boscherberg (48, 1) |
| NH080 | 2 | Napier (55, 2) |
| NH081 | 1 | Riversdale (56, 1) |
| NH082 | 1 | Porterville (50, 1) |
| NH083 | 1 | Grabouw (53, 1) |
| **Lineage B2** |  | **5** |
| NH084 | 2 | Mount Currie Nature Reserve (58, 2) |
| NH085 | 2 | Andries Vosloo Kudu Reserve (57, 2) |
| NH086 | 1 | Andries Vosloo Kudu Reserve (57, 1) |
| **Lineage F** |  | **3** |
| NH087 | 2 | Ongeluksnek Nature Reserve (60, 2) |
| NH088 | 1 | Burgersfort (59, 1) |
| **Lineage A5** |  | **4** |
| NH089 | 1 | Botswana: Kasane (8, 1) |
| NH090 | 2 | Fouriesburg (61, 2) |
| NH091 | 1 | Fouriesburg (61, 1) |
| **Lineage A4** |  | **8** |
| NH092 | 1 | Newcastle (65, 1) |
| NH093 | 1 | Pongola (64, 1) |
| NH094 | 4 | Ithala Nature Reserve (63, 1), New Castle (65, 3) |
| NH095 | 1 | Swaziland: Matenga Nature Reserve (62, 1) |
| NH096 | 1 | Swaziland: Matenga Nature Reserve (62, 1) |
| **Lineage C** |  | **33** |
| NH097 | 3 | Hotazel (66, 1), Schweizer-Reneke (67, 1), Postmasburg (68, 1) |
| NH098 | 1 | Grootdrink (69, 1) |
| NH099 | 1 | Groblershoop (72, 1) |
| NH100 | 20 | Willem Pretorius Nature Reserve (11, 1), Schweizer-Reneke (67, 12), Vryburg (79, 7) |
| NH101 | 1 | Soetdoring Nature Reserve (71, 1) |
| NH102 | 1 | Schweizer-Reneke (67, 1) |
| NH103 | 1 | Groblershoop (72, 1) |
| NH104 | 1 | Griekwastad (70, 1) |
| NH105 | 2 | Kimberley (13, 1),Griekwastad (70, 1) |
| NH106 | 1 | Boshof (12, 1) |
| NH107 | 1 | Schweizer-Reneke (67, 1) |
| **Lineage G** |  | **82** |
| NH108 | 3 | Vryburg (79, 3) |
| NH109 | 1 | Tosca (73, 1) |
| NH110 | 2 | Schweizer-Reneke (67, 2) |
| NH111 | 2 | Severn (77, 2) |
| NH112 | 2 | Vanzylsrus (76, 1), Kuruman (80, 1) |
| NH113 | 23 | Upington (9, 7), Vorstershoop (74, 3), Stella (75, 1), Vanzylsrus (76, 3), Severn (77, 2), Tswalu Kalahari Reserve (78, 6), Vryburg (79, 1), |
| NH114 | 2 | Upington (9, 1), Vryburg (79, 1) |
| NH115 | 24 | Upington (9, 4), Stella (75, 7), Vanzylsrus (76, 4), Severn (77, 2), Vryburg (79, 2), Kuruman (80, 5) |
| NH116 | 6 | Tosca (73, 4), Stella (75, 1), Vryburg (79, 1) |
| NH117 | 1 | Stella (75, 1) |
| NH118 | 5 | Stella (75, 2), Vanzylsrus (76, 1), Vryburg (79, 2) |
| NH119 | 11 | Schweizer-Reneke (67, 5), Vanzylsrus (76, 6) |
| **Lineage E** |  | **25** |
| NH120 | 3 | Gibeon (82, 2), Bergville (90, 1) |
| NH121 | 2 | Gelukspruit (88, 2) |
| NH122 | 2 | Kakamas (89, 2) |
| NH123 | 4 | Augrabies (15, 1), Kakamas (89, 3) |
| NH124 | 1 | Dwesa Nature Reserve (91, 1) |
| NH125 | 5 | Keetmanshoop (83, 1), Karasburg (85, 2), Ais-Ais (86, 1), Noenieput (87, 1) |
| NH126 | 1 | Upington (9, 1) |
| NH127 | 1 | Askham (84, 1) |
| NH128 | 1 | Keetmanshoop (83, 1) |
| NH129 | 4 | Upington (9, 2), Augrabies (15, 1), Askham (84, 1) |
| NH130 | 1 | Windhoek (81, 1) |
| **Lineage A1** |  | **1** |
| NH131 | 1 | Koppies Dam Nature Reserve (92, 1) |
| **Lineage A3** |  | **5** |
| NH132 | 1 | Machadodorp (93, 1) |
| NH133 | 1 | Malelane (94, 1) |
| NH134 | 1 | Malelane (94, 1) |
| NH135 | 1 | Machadodorp (93, 1) |
| NH136 | 1 | Malelane (94, 1) |
| **Lineage B1** |  | **1** |
| NH137 | 1 | Volksrust (95, 1) |
