# Supplementary material for: Bioregion heterogeneity correlates with extensive mitochondrial DNA diversity in the Namaqua rock mouse, Micaelamys namaquensis (Rodentia: Muridae) from southern Africa - evidence for a species complex
Source: BMC Evol Biol. 2010 Oct 13;10:307. doi: 10.1186/1471-2148-10-307 (PMC2967545; doi:10.1186/1471-2148-10-307)
Supplement: Additional file 3 — Support for the different lineages using different methods. Support for the different lineages using different methods. "*" indicates resolution and statistical support, "+" indicates resolution but no statistical support, and "-" indicates unresolved. [file 1471-2148-10-307-S3.DOC]

a) Lineages

| Lineages | **A** | **B** | **C** | **D** | **E** | **F** | **G** | **H** |
| --- | --- | --- | --- | --- | --- | --- | --- | --- |
|  |  |  |  |  |  |  |  |  |
| **MrBAYES** | * | + | * | * | * | * | * | - |
| **BEAST** | + | + | + | + | + | + | + | + |
| **TCS** | - | - | + | + | + | + | + | + |
| **ML** | - | - | * | * | * | - | * | - |

b) Sister relationships

| Lineages | **AB** | **CE** | **DE** | **C(DE)/D(CE)** | **(AB)(CDE)** | **F(A-E)** | **G(A-F)** | **H(A-G)** | **GH** |
| --- | --- | --- | --- | --- | --- | --- | --- | --- | --- |
|  |  |  |  |  |  |  |  |  |  |
| **MrBAYES** | + | + | + | + | + | + | * | * | - |
| **BEAST** | + | + | − | + | + | - | - | - | + |
| **ML** | * (+F) | - | * | * | * | - | * | * | - |
