# Supplementary material for: Bioregion heterogeneity correlates with extensive mitochondrial DNA diversity in the Namaqua rock mouse, Micaelamys namaquensis (Rodentia: Muridae) from southern Africa - evidence for a species complex
Source: BMC Evol Biol. 2010 Oct 13;10:307. doi: 10.1186/1471-2148-10-307 (PMC2967545; doi:10.1186/1471-2148-10-307)
Supplement: Additional file 4 — Mantel test for the different haplogroups/lineages within Micaelamys namaquensis . Mantel test results (mitochondrial DNA (mtDNA) cytochrome b (cyt b)) for the different haplogroups/lineages within Micaelamys namaquensis from southern Africa as defined by an allele network and phylogenetic analyses. In most analyses, the standard normal variate (g) was smaller than the critical value of 2.575. See Figures 2, 3, 4 and 5 for the genetic and geographic distinction of the lineages. Mantel test for some lineages were not shown as a result of small sample sizes. P-values in bold indicate significant correlation between geographic and genetic distance. [file 1471-2148-10-307-S4.DOC]

| Groups/lineages as defined by minspnet, tcs and phylogenetic analyses | Standard normal variate (*g*) | Correlation coefficient (r) | *P*-value |
| --- | --- | --- | --- |
| Lineage A2 | 1.97 | 0.18 | 0.027 |
| Lineage A4 | 0.27 | 0.15 | 0.421 |
| Lineage A1-A5 | 4.72 | 0.35 | 0.001 |
| Lineage B3 | 1.36 | 0.31 | 0.145 |
| Lineage B1-B3 | 3.43 | 0.72 | 0.001 |
| Lineage C | -0.42 | -0.07 | 0.395 |
| Lineage D | -0.18 | -0.02 | 0.499 |
| Lineage E | 3.31 | 0.71 | 0.001 |
| Lineage G | -1.14 | -0.20 | 0.156 |
| Lineage H | -0.47 | -0.11 | 0.183 |
