# Supplementary material for: Bioregion heterogeneity correlates with extensive mitochondrial DNA diversity in the Namaqua rock mouse, Micaelamys namaquensis (Rodentia: Muridae) from southern Africa - evidence for a species complex
Source: BMC Evol Biol. 2010 Oct 13;10:307. doi: 10.1186/1471-2148-10-307 (PMC2967545; doi:10.1186/1471-2148-10-307)
Supplement: Additional file 5 — Geographic coordinates of all collecting localities of Micaelamys namaquensis . Geographic coordinates of all collecting localities of Micaelamys namaquensis from southern Africa analysed in the present study. Numbers 1 - 95 correspond to those in Additional file 6. [file 1471-2148-10-307-S5.DOC]

| Locality | Country | Province | Geographic CoordinaTe |
| --- | --- | --- | --- |
| **Lineage H** |  |  |  |
| 1. Farm: Elephant Sands, Nata | Botswana |  | 19°44’56”S 26°04’18”E |
| 2. Francistown, just outside town (municipal grounds) | Botswana |  | 21°11’15”S 27°23’22”E |
| 3. Farm: Terrafou, south of Francistown | Botswana |  | 22°27’29”S 28°45’32”E |
| 4. Musina Nature Reserve, Musina | South Africa | Limpopo | 22°24’45”S 30°03’01”E |
| 5. Marula Lodge Safaris, Alldays | South Africa | Limpopo | 22°35’10”S 29°10’08”E |
| 6. Blouberg Nature Reserve, Vivo | South Africa | Limpopo | 22°59’12”S 29°08’49”E |
| 7. Farm: Goedgelegen, Baltimore | South Africa | Limpopo | 23°26’27”S 28°23’02”E |
| **Lineage D** |  |  |  |
| 8. Kasane, just outside town (municipal grounds) | Botswana |  | 17°47’07”S 25°10’59”E |
| 9. Farm: Steenkampsput, Upington | South Africa | Northern Cape | 28°06’13”S 20°54’10”E |
| 10. Farm: Warmhoek, Hoopstad | South Africa | Free State | 28°10’08”S 25°49’11”E |
| 11. Willem Pretorius Nature Reserve, Winburg | South Africa | Free State | 28°16’27”S 27°14’48”E |
| 12. Farm: Viljoenshof, Boshof | South Africa | Free State | 28°34’45”S 25°04’33”E |
| 13. Langeberg Guest Farm, Kimberley | South Africa | Northern Cape | 28°54’47”S 24°38’33”E |
| 14. Farm: Palmietfontein, Brandfort | South Africa | Free State | 28°48’07”S 26°33’32”E |
| 15. Farm: Tierkoppen, Augrabies | South Africa | Northern Cape | 28°34’06”S 20°26’05”E |
| 16. Jacobsdal Agricultural School, Bloemfontein | South Africa | Free State | 29°10’12”S 26°19’48”E |
| 17. Farm: Boomrivier, Pofadder | South Africa | Northern Cape | 29°04’33”S 19°18’24”E |
| 18. Farm: Rietfontein, Springbok | South Africa | Northern Cape | 29°51’40”S 18°11’10”E |
| 19. Hopetown | South Africa | Northern Cape | 29°44’45”S 23°37’30”E |
| 20. Caledon Nature Reserve, Wepener | South Africa | Free State | 29°49’30”S 26°53’16”E |
| 21. Gariep Nature Reserve, Gariep Dam | South Africa | Free State | 30°35’56”S 25°32’03”E |
| 22. Lady Grey, just outside town (municipal grounds) | South Africa | Eastern Cape | 30°45’00”S 27°15’00”E |
| 23. Farm: Klipfontein, Jamestown | South Africa | Eastern Cape | 31°11’23”S 26°49’12”E |
| 24. Farm: Rietpoort, Loxton | South Africa | Northern Cape | 31°38’30”S 22°22’34”E |
| 25. Karoo National Park, Beaufort West | South Africa | Northern Cape | 32°15’00”S 22°30’00”E |
| 26. Matjiesfontein, just outside town (municipal grounds) | South Africa | Western Cape | 33°15’00”S 20°34’48”E |
| 27. Farm: Brakrivier, Oudtshoorn | South Africa | Western Cape | 33°46’19”S 22°31’45”E |
| 28. Kirkwood, just outside town (municipal grounds) | South Africa | Eastern Cape | 33°24’20”S 25°25’30”E |
| **Lineage A2** |  |  |  |
| 29. Lajuma Mountain Retreat, Makhado | South Africa | Limpopo | 23°02’02”S 29°26’27”E |
| 30. Ellisras | South Africa | Limpopo | 23°40’12”S 28°45’00”E |
| 31. Lapalala Nature Reserve, Vaalwater | South Africa | Limpopo | 23°52’04”S 28°19’55”E |
| 32. Amanita Safaris, Rooibokkraal | South Africa | Limpopo | 24°09’16”S 26°55’05”E |
| 33. Ben Alberts Nature Reserve, Thabazimbi | South Africa | Limpopo | 24°34’48”S 27°25’12”E |
| 34. Farm: Waterval, Thabazimbi | South Africa | Limpopo | 24°31’12”S 27°45’00”E |
| 35. Gaborone, just outside town (municipal grounds) | Botswana |  | 24°40’12”S 25°49’48”E |
| 36. Farm: Sunset Ranch, Bela-Bela | South Africa | Limpopo | 24°45’00”S 28°15’00”E |
| 37. Selati Nature Reserve, Hoedspruit | South Africa | Limpopo | 24°09’30”S 30°40’50”E |
| 38. Farm: Boskloof, Boshoek | South Africa | North West | 25°28’43”S 27°03’39”E |
| 39. Kgaswane Mountain Reserve, Rustenburg | South Africa | North West | 25°44’20”S 27°12’56”E |
| 40. Brits Agricultural School, Brits | South Africa | North West | 25°34’29”S 27°46’02”E |
| 41. Ezemvelo Nature Reserve, Bronkhorstspruit | South Africa | Gauteng | 25°45’00”S 28°49’48”E |
| 42. Kruisrivier Nature Reserve, Loskop Dam | South Africa | Mpumalanga | 25°21’08”S 29°32’26”E |
| 43. Farm: Rietfontein, Potchefstroom | South Africa | North West | 26°38’36”S 27°21’48”E |
| 44. Farm: Ratzegaai, Ventersdorp | South Africa | North West | 26°20’30”S 26°44’01”E |
| 45. Habula Lodge, Vredefort | South Africa | Free State | 26°53’48”S 27°19’20”E |
| 46. Josefsdal Nature Reserve, Barberton | South Africa | Mpumalanga | 25°58’05”S 30°42’57”E |
| 47. Farm: Uitspanning, Amsterdam | South Africa | Mpumalanga | 26°39’56”S 30°31’26”E |
| **Lineage B3** |  |  |  |
| 48. Boscherberg, Algeria, Cederberg | South Africa | Western Cape | 32°10’10”S 19°04’05”E |
| 49. Jamaka, Algeria, Cederberg | South Africa | Western Cape | 32°20’20”S 19°05’05”E |
| 50. Farm: Grootfontein, Porterville | South Africa | Western Cape | 32°54’28”S 19°06’31”E |
| 51. Vrolijkheid Nature Reserve, Jonaskop | South Africa | Western Cape | 33°45’10”S 19°30’10”E |
| 52. Farm: Goederede, Robertson | South Africa | Western Cape | 33°45’45”S 19°40’20”E |
| 53. Farm: Mizpah, Grabouw | South Africa | Western Cape | 34°10’10”S 19°02’15”E |
| 54. Vrolijkheid Nature Reserve, Die Galg | South Africa | Western Cape | 34°10’10”S 19°55’10”E |
| 55. Farm: Fairfield, Napier | South Africa | Western Cape | 34°27’27”S 19°45’10”E |
| 56. Farm: Versig, Riversdale | South Africa | Western Cape | 34°10’20”S 21°15’15”E |
| **Lineage B2** |  |  |  |
| 57. Andries Vosloo Kudu Reserve, Grahamstown | South Africa | Eastern Cape | 33°10’55”S 26°38’10”E |
| 58. Mount Currie Nature Reserve, Kokstad | South Africa | KwaZulu-Natal | 30°29’36”S 29°23’18”E |
| **Lineage F** |  |  |  |
| 59. Gethlane Lodge, Burgersfort | South Africa | Mpumalanga | 24°45’51”S 30°23’11”E |
| 60. Ongeluksnek Nature Reserve, Thaba Chitja | South Africa | Eastern Cape | 30°20’05”S 28°21’17”E |
| **Lineage A5** |  |  |  |
| 61. Wynford Guest Farm, Fouriesburg | South Africa | Free State | 28°30’30”S 28°15’42”E |
| **Lineage A4** |  |  |  |
| 62. Mantenga Nature Reserve | Swaziland |  | 26°26’37”S 31°10’22”E |
| 63. Ithala Nature Reserve, Louwsburg | South Africa | KwaZulu-Natal | 27°30’10”S 31°15’10”E |
| 64. Farm: Koedoesberg, Pongola | South Africa | KwaZulu-Natal | 27°26’31”S 31°41’41”E |
| 65. Newcastle | South Africa | KwaZulu-Natal | 28°04’22”S 29°48’02”E |
| **Lineage C** |  |  |  |
| 66. Farm: Karlsrühe, Hotazel | South Africa | Northern Cape | 26°58’34”S 22°59’57”E |
| 67. Farm: Donkerpoort, Schweizer-Reneke | South Africa | North West | 27°14’46”S 25°06’01”E |
| 68. Farm: Tierkop, Postmasburg | South Africa | Northern Cape | 28°21’33”S 23°14’33”E |
| 69. Farm: Swemkuil, Grootdrink | South Africa | Northern Cape | 28°39’07”S 21°47’54”E |
| 70. Witsand Nature Reserve, Griekwastad | South Africa | Northern Cape | 28°43’52”S 22°26’08”E |
| 71. Soetdoring Nature Reserve, Bloemfontein | South Africa | Northern Cape | 23°50’50”S 26°08’55”E |
| 72. Farm: Rooidam, Groblershoop | South Africa | Northern Cape | 29°08’33”S 22°19’34”E |
| **Lineage G** |  |  |  |
| 73. Farm: Welbedeur, Tosca | South Africa | North West | 25°42’53”S 23°58’43”E |
| 74. Farm: Arizona, Vorstershoop | South Africa | North West | 25°57’00”S 23°13’55”E |
| 75. Farm: Rus en Vrede, Stella | South Africa | North West | 26°10’23”S 25°13’27”E |
| 76. Farm: Loversleap, Vanzylsrus | South Africa | Northern Cape | 26°38’20”S 22°01’4”E |
| 77. Farm: Jones, Severn | South Africa | Northern Cape | 26°35’22”S 22°41’46”E |
| 78. Tswalu Kalahari Reserve, Sonstraal | South Africa | Northern Cape | 27°12’51”S 22°’27’22”E |
| 79. Farm: Waterloo & Vlakfontein, Vryburg | South Africa | North West | 27°03’34”S 24°45’58”E |
| 80. Farm: Strelley, Kuruman | South Africa | Northern Cape | 27°39’48”S 23°23’04”E |
| **Lineage E** |  |  |  |
| 81. Windhoek | Namibia |  | 22°35’32”S 17°10’26”E |
| 82. Gibeon | Namibia |  | 25°20’42”S 17°15’13”E |
| 83. Quivertree Forest Rest Camp, Keetmanshoop | Namibia |  | 26°28’56”S 18°14’39”E |
| 84. Farm: Koppieskraal, Askham | South Africa | Northern Cape | 26°56’18”S 20°13’38”E |
| 85. Farm: Duurdrift, Karasburg | Namibia |  | 27°26’10”S 18°53’17”E |
| 86. Canon Lodge, Ais-Ais | Namibia |  | 27°39’49”S 17°46’42”E |
| 87. Farm: Witkoppen, Noenieput | South Africa | Northern Cape | 27°35’44”S 20°13’49”E |
| 88. Farm: Swartmodder, Gelukspruit | South Africa | Northern Cape | 28°01’45”S 20°33’33”E |
| 89. Farm: Zwartbooisberg, Kakamas | South Africa | Northern Cape | 28°02’30”S 20°42’55”E |
| 90. Farm: Meedwood, Bergville | South Africa | KwaZulu-Natal | 28°42’44”S 29°19’25”E |
| 91. Dwesa Nature Reserve, Dutywa | South Africa | Eastern Cape | 32°18’02”S 28°49’40”E |
| **Lineage A1** |  |  |  |
| 92. Koppies Dam Nature Reserve, Koppies | South Africa | Free State | 27°13’27”S 27°40’29”E |
| **Lineage A3** |  |  |  |
| 93. Wathaba-Uitkomst, Machadodorp | South Africa | Mpumalanga | 25°47’31”S 30°22’28”E |
| 94. Farm: Riverside, Malelane | South Africa | Mpumalanga | 25°26’33”S 31°33’01”E |
| **Lineage B1** |  |  |  |
| 95. Farm: Waterval, Volksrust | South Africa | Mpumalanga | 27°22’55”S 29°45’31”E |
