# Supplementary figures and images for: Bioregion heterogeneity correlates with extensive mitochondrial DNA diversity in the Namaqua rock mouse, Micaelamys namaquensis (Rodentia: Muridae) from southern Africa - evidence for a species complex
Source: BMC Evol Biol. 2010 Oct 13;10:307. doi: 10.1186/1471-2148-10-307 (PMC2967545; doi:10.1186/1471-2148-10-307)

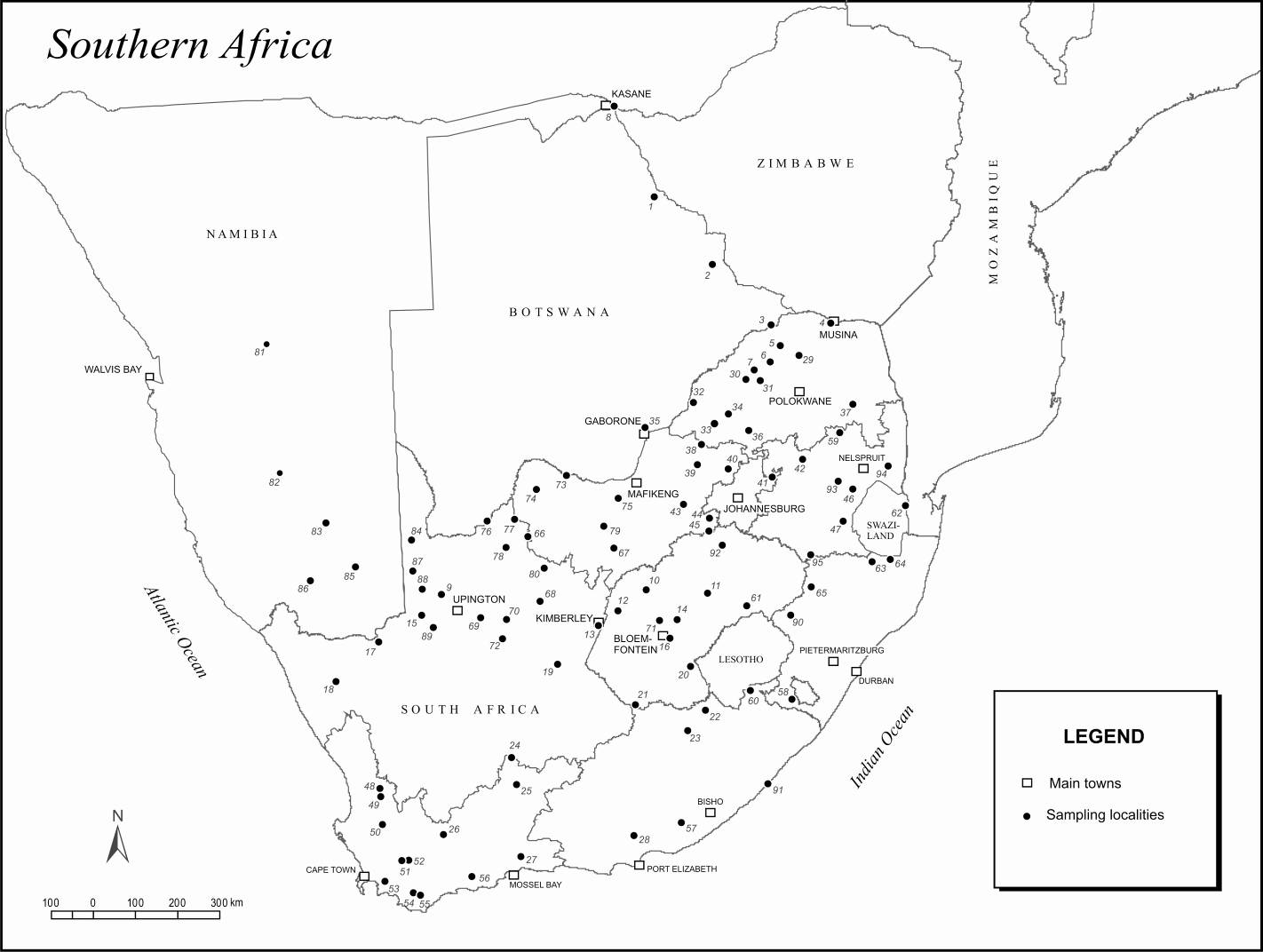

Supplement: Additional file 6 — Collecting localities of samples of Micaelamys namaquensis . Collecting localities of samples of Micaelamys namaquensis from southern Africa. Numbers correspond to the locality numbers and names in Additional file 5. [file 1471-2148-10-307-S6.DOC]
