# Supplementary material for: Bioregion heterogeneity correlates with extensive mitochondrial DNA diversity in the Namaqua rock mouse, Micaelamys namaquensis (Rodentia: Muridae) from southern Africa - evidence for a species complex
Source: BMC Evol Biol. 2010 Oct 13;10:307. doi: 10.1186/1471-2148-10-307 (PMC2967545; doi:10.1186/1471-2148-10-307)
Supplement: Additional file 7 — Permits and permit numbers. Permits and permit numbers for the nine provinces representing South Africa and permits for Botswana, Swaziland and Namibia. [file 1471-2148-10-307-S7.DOC]

| Province | Permit number | Permit holder |
| --- | --- | --- |
| Free State | HK/P1/07106/001 | I.M. Russo |
| Gauteng | 1244 | I.M. Russo |
| Mpumalanga | MPB. 5126 | I.M. Russo |
| Eastern Cape | Letter with no permit number | I.M. Russo |
| Limpopo | CMP-004-00004 | I.M. Russo |
| Kwa-Zulu Natal | 3968/2004 | I.M. Russo |
| Northern Cape | 040/2001 | I.M. Russo |
| Northern Cape | 0545/2004 | I.M. Russo |
| North West | 000027 NW-06 | I.M. Russo |
| Western Cape | 378/2003 | I.M. Russo |
| Cape Peninsula National Park | Letter with no permit number | I.M. Russo |
| Namibia | 804/2004 | I.M. Russo |
| Swaziland | Letter with no permit number | I.M. Russo |
| Botswana | 13/1/1/30/1-86 | N. Maputla |
